# The genus *Entomophthora*: bringing the insect destroyers into the twenty-first century

**DOI:** 10.1186/s43008-021-00084-w

**Published:** 2021-11-12

**Authors:** Carolyn Elya, Henrik H. De Fine Licht

**Affiliations:** 1 Department of Organismic and Evolutionary Biology, Harvard University, Cambridge, MA USA; 2 Department of Plant and Environmental Sciences, University of Copenhagen, 1871 Frederiksberg, Denmark

**Keywords:** *Entomophthorales*, *Zoopagomycota*, Early-diverging fungi, Entomopathogens, Fungal pathogens, Behavioral manipulation, Insect–fungus interactions, Coevolution

## Abstract

The fungal genus *Entomophthora* consists of highly host-specific pathogens that cause deadly epizootics in their various insect hosts. The most well-known among these is the “zombie fly” fungus *E. muscae*, which, like other *Entomophthora* species, elicits a series of dramatic behaviors in infected hosts to promote optimal spore dispersal. Despite having been first described more than 160 years ago, there are still many open questions about *Entomophthora* biology, including the molecular underpinnings of host behavior manipulation and host specificity. This review provides a comprehensive overview of our current understanding of the biology of *Entomophthora* fungi and enumerates the most pressing outstanding questions that should be addressed in the field. We briefly review the discovery of *Entomophthora* and provide a summary of the 21 recognized *Entomophthora* species, including their type hosts, methods of transmission (ejection of spores after or before host death), and for which molecular data are available. Further, we argue that this genus is globally distributed, based on a compilation of *Entomophthora* records in the literature and in online naturalist databases, and likely to contain additional species. Evidence for strain-level specificity of hosts is summarized and directly compared to phylogenies of *Entomophthora* and the class *Insecta*. A detailed description of *Entomophthora*’s life-cycle and observed manipulated behaviors is provided and used to summarize a consensus for ideal growth conditions. We discuss evidence for *Entomophthora*’s adaptation to growth exclusively inside insects, such as producing wall-less hyphal bodies and a unique set of subtilisin-like proteases to penetrate the insect cuticle. However, we are only starting to understand the functions of unusual molecular and genomic characteristics, such as having large > 1 Gb genomes full of repetitive elements and potential functional diploidy. We argue that the high host-specificity and obligate life-style of most *Entomophthora* species provides ample scope for having been shaped by close coevolution with insects despite the current general lack of such evidence. Finally, we propose six major directions for future *Entomophthora* research and in doing so hope to provide a foundation for future studies of these fungi and their interaction with insects.

**Supplementary Information:**

The online version contains supplementary material available at 10.1186/s43008-021-00084-w.

## INTRODUCTION

Species in the genus *Entomophthora* are fungal pathogens of a variety of insects, most of which elicit dramatic behavioral changes in their host for the invading fungus’ benefit. The genus *Entomophthora* belongs to the early-diverging subphylum *Entomophthoromycotina*,^1^ which includes fungi such as the genera *Basidiobolus* and *Conidiobolus* that can cause a wide range of infections and complications in invertebrates, cold-blooded animals and even humans. The first *Entomophthora* species was formally described in the mid-nineteenth century and these fungi are commonly observed around the world, yet details of their biology have remained mostly a mystery. Recent studies of *E. muscae*, including genomic and transcriptomic analyses as well as the isolation of a strain that naturally infects the model organism *Drosophila melanogaster (E. muscae* isolate ‘Berkeley’*)*, have exposed a new generation of scientists to these unique fungi and sparked renewed interest in their study. This review aims to distill information that is dispersed over a variety of not-so-easily accessed sources (including books, folios and non-English sources) to provide a comprehensive overview of the biology of all known species within the genus *Entomophthora.* In revisiting what we have learned over the past century and a half in combination with recent developments providing new genomic and molecular insights, we hope to provide an accessible entry point for new researchers interested in these incredible fungi, as well as remind those in the field of important gaps in our knowledge and suggest ways to continue moving the field forward into the twenty-first century.

We start by briefly discussing the initial discovery of *Entomophthora* fungi and history of its early research, then present currently accepted species, who they infect and where to find them. Though the interest of this review is not to take a deep dive into fungal systematics, phylogeny and identification, a brief discussion of these topics at the outset is necessary to put these fungi and their corresponding literature into context. We then discuss what is known about the life-cycle, including their effects on host behavior, before transitioning to discuss what we know about the molecular and cell biology of these pathogens. Finally, we will consider the many exciting avenues for future *Entomophthora* research in a variety of biological subdisciplines.

## IINITIAL DISCOVERY TO THE MODERN ERA

“Everyone is familiar with the peculiar way of death of the common house fly...The beginning of the disease is not manifested externally by any special characteristics…but has the disease of the flies reached its last stage, then their movements are extremely sluggish, and when one approaches them they do not fly up at all...About an hour before death all locomotion ceases; the animal sucks itself tight with its proboscis; the legs alone twitch...The abdomen swells more and more and has a very clear white color...Gradually the movements of agony cease; the animal no longer reacts to external stimuli. After death, the abdomen continues to swell...a white substance pushes out between them...On the ground you notice the first touch of dust...the three wide bands become wider and higher...at the same time the mass of dust increases steadily. Gradually the body dries up, the white rings disappear, the stretched body shrinks...and the fly almost assumes its usual appearance...but the wings and legs remain covered with dust.”

(Ferdinand Cohn (Cohn 1855), translated from the German).

**Fig. 1:**
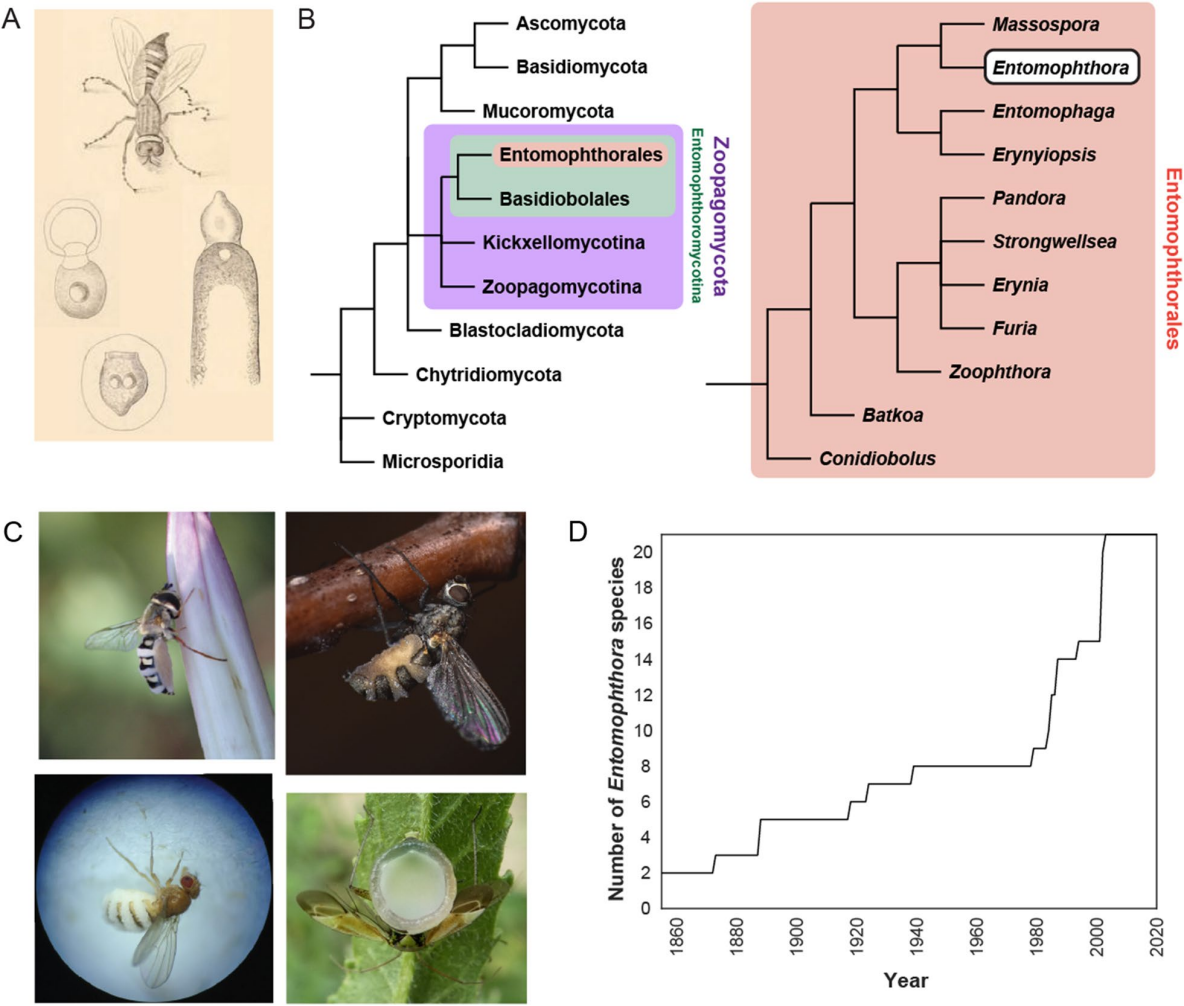
What is *Entomophthora*? **A** Early camera lucida drawings of *E. muscae* (Cohn 1855). Clockwise from top: House fly killed by *E. muscae*, conidiophore forming a primary conidium, ejected primary conidium surrounded by cytoplasmic halo, primary conidium giving rise to secondary conidium. **B** (Left) Schematic fungal cladogram based on (James et al. 2013; Spatafora et al. 2016); branch lengths are not proportional to genetic distances; the phylum *Zoopagomycota* encompasses the division *Entomophthoromycotina*, which in turn contains the order *Entomophthorales*. (Right) Schematic cladogram of order *Entomophthorales* based on (Gryganskyi et al. 2012); the position of *Entomophthora* is highlighted near the top. **C** Insects killed by fungi in the genus *Entomophthora*. Clockwise from top left: syrphid killed by *E. syrphi*, muscoid killed by *E. muscae*, mirid killed by *E. erupta*, *Drosophila melanogaster* killed by *E. muscae* isolate ‘Berkeley’. Images provided under CC BY-NC license credits by iNaturalist users silverseastarsong (James Bailey), xx7trey (Trey Wardlaw) and dlbowls, respectively. Bottom left image provided by Carolyn Elya. **D** Number of currently recognized *Entomophthora* species over time

*Entomophthora* is a genus of obligate insect pathogens within the early-diverging fungal phylum *Zoopagomycota* (formerly *Zygomycota*) (Spatafora et al. 2016) (Fig. 1). The name is fitting, coming from the Greek “*Entomo*” meaning insect and “*phthora*” meaning destroyer, as these fungi infect and ultimately consume their insect hosts, in many cases modifying end-of-life behavior in the host to aid in spore dispersal. The first species to be formally reported in the scientific literature was *Entomophthora muscae* (originally called *Empusa muscae*^2^), described as above in 1855 by Cohn (1855). Commenting that the fungus is “one of the strangest and most interesting apparitions”, he provided a detailed description of the fungus based on his observation of fungus-filled flies adhered to the drapes in his home in Germany. From Cohn’s publication, it is clear that he was not the first to ever observe the fungus, just the first to record his observations at length: “That this strange way of death of the flies, which is known to every child, escaped only natural scientists, is not to be assumed…”

The end of the nineteenth century saw the description of a variety of entomophthoralean fungi in American and European literature (Braun 1855, 1856; Brefeld 1870, 1871, 1877; Cornu 1873; Giard 1888; Thaxter 1888). Over the next hundred years, dozens of new species designated as *Entomophthora* (and *Empusa*) continued to be described in a variety of insects in both the United States and Europe (MacLeod and Müller-Kögler 1973; MacLeod et al. 1976). Species were reported as novel on the basis of slight morphological differences from species previously described, difference in host species, or location found (MacLeod and Müller-Kögler 1973; MacLeod et al. 1976). While there is much value in revisiting early publications in this field, especially for the beautiful camera lucida drawings of several life stages and detailed natural history descriptions (e.g., Fig. 1A), a high degree of caution should be exercised in interpreting the reported species name at face value. For example, a publication as late as 1964 claimed to determine a method for growing *Entomophthora muscae* mycelium on a simple medium—a feat that had been attempted many times on a variety of bizarre media (e.g., lard, asparagus, butter; Güssow 1917), before but never achieved. However, this was later found to not have been *E. muscae* at all, but likely a *Conidiobolus* species (Srinivasan et al. 1964). In addition, while many species described in the twentieth century were initially designated as members of *Entomophthora*, additional morphological characterization and revision of genus definitions eventually led them to be assigned to other entomophthoralean genera (e.g., *Conidiobolus*, *Entomophaga*, *Erynia*, *Eryniopsis*, *Zoophthora*, *Furia*, and *Pandora* (Remaudiere and Keller 1980)), designations which have since been supported by molecular phylogenetic analysis (Gryganskyi et al. 2012). As an example of the degree of taxonomic flux in this field, a 1963 survey of *Entomophthora* in the Western Hhemisphere presented data for 39 species, only three of which (*E. muscae*, *E. erupta,* and *E. culicis*) are still recognized as belonging to the genus today (Hutchison 1963).

Owing largely to the high degree of morphological similarity between many species and varied interpretations of which morphological features are most important for separating species, disagreement about taxonomy of entomophthoralean fungi and what constituted the genus *Entomophthora* continued until 1980 (Macleod 1963; Batko and Weiser 1965). Finally, it was proposed that all fungi that forcibly discharge campanulate (bell-shaped) primary conidia should be considered *Entomophthora*: this remains the accepted definition of the genus (Remaudiere and Keller 1980).

## WHO THEY ARE, WHERE TO FIND THEM, AND WHO THEY KILL

As of this writing, there are 21 species of *Entomophthora* recognized in the literature, most (possibly all) of which elicit behavior changes in their host that promote spore dispersal (Table 1). Species boundaries for these and other entomophthoralean fungi are currently delineated based on a combination of morphology of different growth stages (usually number of nuclei and dimensions of primary conidia; Fig. 5), the host in which the fungus was observed and where geographically it was found, usually in that order (e.g., Keller 2007). There are already several publications that comprehensively detail the morphology and taxonomy of *Entomophthora* (Humber 1984, 1989, 2012a, b, 2016; Samson et al. 1988; Keller 2007), so these details will not be recounted here. While molecular data for conserved loci are available for some isolates (e.g., internal transcribed spacer [ITS], and small and/or large ribosomal rRNA), sequencing data has not been collected for many described species. As we discuss later, genomic sequencing of *Entomophthora* species is more challenging than for many other described fungi, and this challenge has played a large role in stalling the transition to molecular-based taxonomy.

**Table 1:** Recognized *Entomophthora* species

Species	First description	Type host^2^	Spore dispersal^4^	Presence in GenBank^5^	Deposited in ARSEF^6^	Altered behavior^7^
*E. brevinucleata*^1^	Keller and Wilding (1985)	*Sitodiplosis phalaridis* (Gall midge)	CT			X
*E. byfordii*	Keller (2002)	*Bradysia* sp. (Fungus gnat)	CT	X		X
*E. chromadphidis*	Burger and Swain (1918)	*Chromaphidis juglandicola* (Walnut aphid)	CT	X	X	
*E. culicis*	Braun (1855)	*Culex pipiens* (House mosquito)	CT	X	X	X (Gol’berg 1979)
*E. erupta*	Dustan (1924)	*Lygus communis* (Tarnished plant bug)	AHT			X
*E. ferdinandii*	Keller (2002)	*Delia kullensis* (Anthonymiid fly)	CT	X	X	X
*E. grandis*	Keller (2002)	*Episyrpho balteato* (Hoverfly)	CT	X		X
*E. helvetica*	Ben-Ze’ev' et al. 1(985)	*Notostira elongata* (Mirid)	CT			X
*E. israelensis*	Ben-Ze’ev and Zelig (1984)	Gall midges	CT			X
*E. leyteensis*	(Villacarlos et al. 2003)	*Tetraleurodes acaciae* (Whitefly)	CT			X
*E. muscae*	Cohn (1855)	*Musca domestica* (House fly)	CT	X	X	X
*E. philippinensis*	Villacarlos and Wilding (1994)	*Heteropsylla cubana* (Jumping louse)	CT			X
*E. planchoniana*	Cornu (1873)	*Aphis sambuci*^*3*^ (Elder aphid)	CT	X	X	
*E. rivularis*	Keller (2002)	*Plecoptera* sp. (Stoneflies)	CT			
*E. scatophagae*	Giard (1888)	*Scatophaga stercoraria* (Golden dung fly)	CT	X	X	X
*E. schizophorae*	Keller (1987)	*Delia platura* (Bean seed fly)	CT	X	X	X
*E. simulii*	Keller (2002)	*Simulium lineato* (Blackfly)	CT			X
*E. syrphi*	Giard (1888)	*Melanostoma mellinum* (Hoverfly)	CT	X	X	X
*E. thripidum*	Samson et al. (1979)	*Thrips tabaci* (Onion thrips)	AHT	X	X	X
*E. trinucleata*	Keller (1987)	*Sciaridae* sp. (Dark-wing fungus gnat)	CT			X
*E. weberi*	Lakon (1939)	*Raphidia ophiopsis* (Snakefly larvae)	AHT			X

Underlined species are members of the *E. muscae* species complex per Keller 1984 and Humber 1989. An alternative assessment of the *E. muscae* species complex includes these four species plus *E. brevinucleata*, *E. israelensis, E. syrphi* and *E. trinucleata* (Keller 1984; Humber 1989)

^1^This species has been reported as synonymous with *E. israelensis* (Humber 1989), but was given as a distinct species in Keller (2002)

^2^The most specific designation of type host is given, according to (Keller 2002)

^3^Presumed type host based on original description (Keller 2002)

^4^AHT = active host transmission; CT = cadaver transmission

^5^Presence in GenBank indicates that at least one sequence annotated with indicated species is present in GenBank (National Institute of Health sequence database, https://www.ncbi.nlm.nih.gov/genbank/). Deposited sequences mostly consist of ITS and rRNA loci, with additional gene sequences available for E. muscae

^6^USDA Agricultural Research Service Collection of Entomopathogenic Fungal Cultures, https://www.ars.usda.gov/

^7^X indicates reported altered end-of-life behavior; blank indicates absence of evidence. As rigorous behavioral studies have not taken place in most species, we are inferring behavior modification from death position/stance or aberrant location of corpses (i.e., dead insects where they are not typically found if killed by other means). Absence of evidence for behavior modification does not preclude more subtle behavioral changes that are not conspicuous to the human eye. Reports of altered end-of-life behavior can be found in the first publication describing the species (“First description”), unless where otherwise noted

**Fig. 2:**
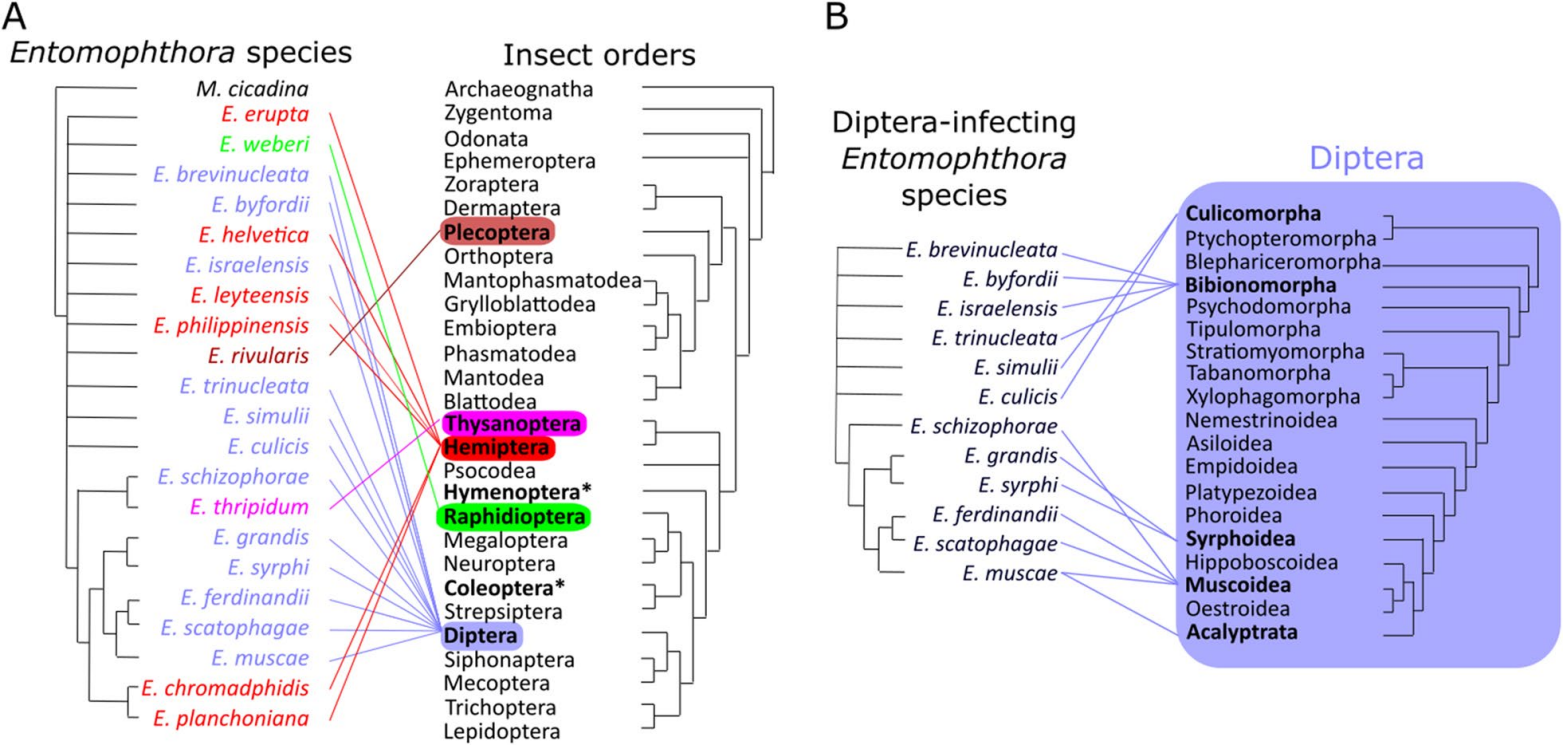
Potential coevolution and host ranges of *Entomophthora.* **A** Schematic co-cladogram of 21 recognized *Entomophthora* species and orders within the class *Insecta*; *Entomophthora* species in blue text all infect *Diptera*, red *Hemiptera*, green *Raphidoptera*, pink *Thysanoptera*, and dark red *Plecoptera*; the asterisk (*) highlights the orders *Hymenoptera* and *Coleoptera* with known infections of undescribed *Entomophthora* species (Eilenberg et al. 1987). **B** Schematic co-cladogram of fly-infecting *Entomophthora* species and major families/superfamilies within *Diptera*; phylogenetic relationships of *Entomophthora* species are based on Gryganskyi et al. (2013a), insect orders from Misof et al. (2014), and *Diptera* families/superfamilies from Wiegmann et al. (2011)

The hosts of *Entomophthora* include species from the orders: *Diptera* (true flies), *Hemiptera* (true bugs), *Raphidioptera* (snakeflies), *Plecoptera* (stoneflies) and *Thysanoptera* (thrips) (Keller 2007), insects that last shared a common ancestor around 400 million years ago in the Devonian period (Misof et al. 2014) (Fig. 2). The most recent multi-locus phylogeny of *Entomophthoromycota* predicts that the ancestor of obligate entomophthoralean insect pathogens arose 225 ± 75 Mya (Gryganskyi et al. 2012). Considering that this estimate is based on just a handful of loci and likely to change with additional genetic data, it seems likely that the last common ancestor of these fungi was also an obligate parasite of a Devonian insect host.

**Fig. 3:**
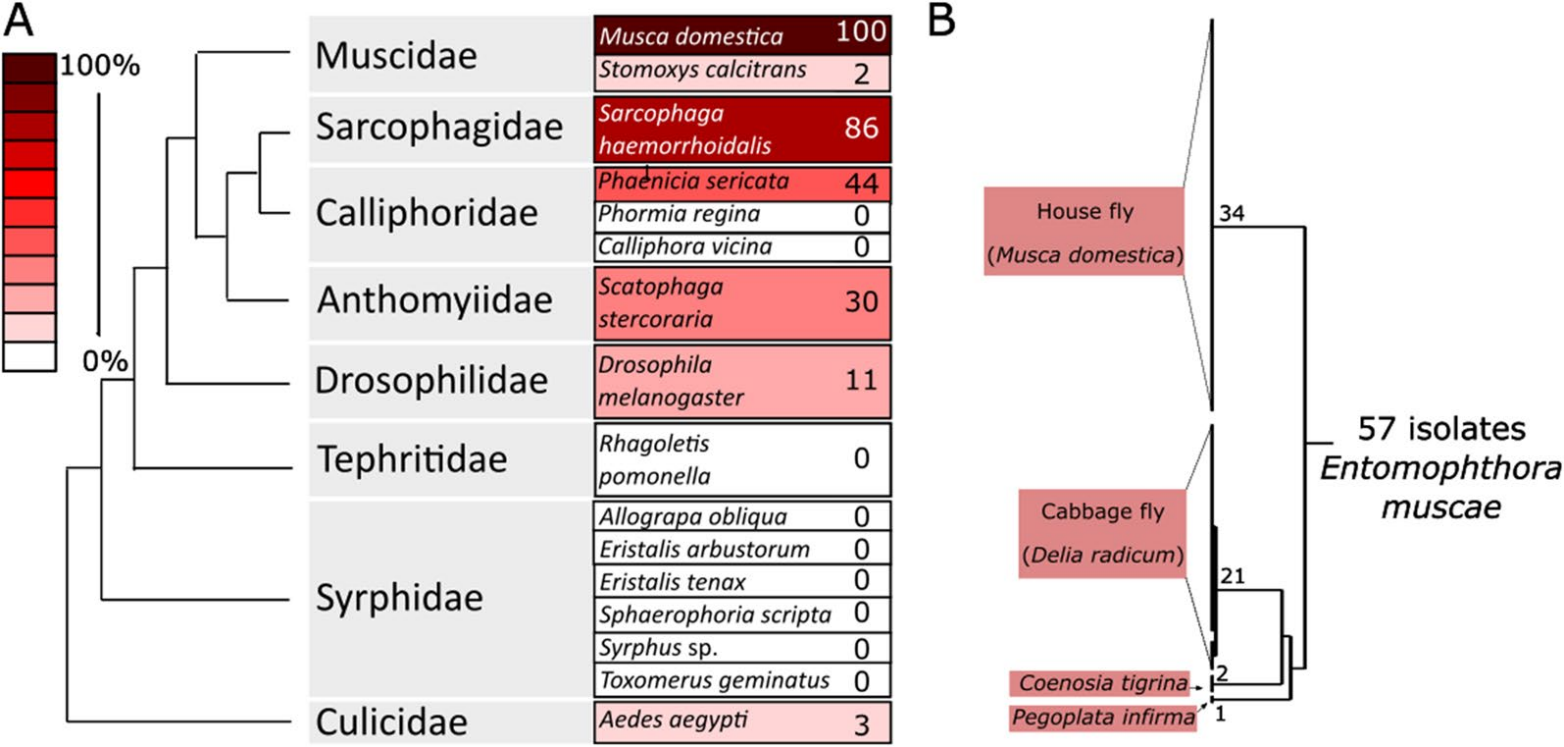
Host specificity of *Entomophthora muscae* from house flies (*Musca domestica*). **A** One *E. muscae* isolate from house flies (*Musca domestica*) were experimentally exposed to 16 different insect species by Steinkraus & Kramer (Steinkraus and Kramer 1987); the numbers and red heatmap depicts percentage successful infections showing 100% infections in the natural host and varying infection success in other species; *S. calcitrans* and *A. aegypti* showed atypical infections with very limited conidia production (Steinkraus and Kramer 1987). **B** Schematic drawing of genetic differentiation of 57 *E. muscae* isolates based on RAPD markers (Jensen and Eilenberg 2001). Branch lengths not drawn to scale in B, and node markings refer to number of genetically similar *E. muscae* isolates within that clade

The host in which the fungal species was first formally described is referred to here as the type host, though it is worth emphasizing that a given fungal species (as currently defined) may naturally infect species other than its type host (Fig. 3). While for many *Entomophthora* species, host range appears to be narrow, species of *Entomophthora* with morphology indistinguishable to or overlapping that of *E. muscae* have been observed to infect a broad range of dipteran hosts (Fig. 3). However, several observations have been made that support the idea that *E. muscae* is not a homogeneous species, but rather a species complex, a group of multiple species that cannot be distinguished on morphology alone (Keller 1984).

First, a series of studies has found that isolates from different hosts, while morphologically very similar, show differing patterns in restriction fragment length polymorphism (RFLP) and random amplified polymorphic DNA (RAPD) assays (Jensen and Eilenberg 2001; Jensen et al. 2001, 2006). This indicates a high degree of molecular heterogeneity that generally tracks with host identity (Fig. 3). Also, naturally-occurring outbreaks of *E. muscae* infection appear to target specific host species. Keller observed that in an ongoing *E. muscae* outbreak in a stable, only *Musca domestica* were observed to die of fungal infection and sporulate, even though 40% of the fly population in the stable was made up by another dipteran species, *Stomoxys calcitrans* (Keller 2002). While some *S. calcitrans* individuals were found dead in the stable, none produced conidia. Additionally, a 2013 study reported an epizootic event that first predominantly affected *Delia radicum* then shifted to mostly affect *Coenosia tigrina* (Gryganskyi et al. 2013b). Targeted locus sequencing of flies infected during this outbreak revealed the presence of two different fungal haplotypes, one mostly found in *D. radicum* and the other in *C. tigrina*, though there were a few instances where the haplotypes were found in the less common host. Again, though several fly species other than *D. radicum* and *C. tigrina* were observed in the area of this outbreak, only those two species were ever observed to be killed by *E. muscae*. With the acquisition of more molecular data and clarification of the diversity of these fungi, it seems likely that we will find that what we now refer to as *E. muscae* is actually a collection of morphologically indistinguishable species, i.e., cryptic species. Such a finding would be consistent with the generally accepted idea that the specificity of the behavior manipulations induced by these fungi reflects intense specialization, which would be expected to come at the cost of generality (Schmid-Hempel 2011).

Due to the long-sought efforts to employ *Entomophthora* spp*.* as biocontrol agents (Brongniart 1888; Brumpt 1941), studies have also found various fungal species capable of infecting hosts that have not been observed to be naturally infected in the wild. For example, *E. culicis* has been shown to infect and kill *Aedes aegypti* mosquitoes (Kramer 1982), *E. muscae* has been found to infect and kill a diverse panel of 16 dipteran species in the laboratory including *Anopheles* mosquitoes (Kramer and Steinkraus 1981; Steinkraus and Kramer 1987) (Fig. 3), and it was possible to infect house flies (*Musca domestica*) with an undescribed *Entomophthora* sp. found on a beetle (*Coleoptera*; Eilenberg et al. 1987). While *Entomophthora* spp*.* may be able to infect and kill species that have not yet been observed to be naturally infected, these fungi are not always capable of manipulating the behavior of these foreign hosts or producing the spores needed to infect subsequent victims (Fig. 3). Even if a particular fungal species is shown to be capable of infecting a novel host in the laboratory, one should be cautious in extrapolating what is possible experimentally to what happens in a natural setting. First, aspects of host ecology and/or physiology may preclude it from ever becoming infected under natural conditions. As mentioned previously, observations have been made of *E. muscae* infecting one species in the context of multiple potential hosts. This would suggest that even if species exist in the same environment, factors such as differences in behavior, preferred substrates and/or fungal specificity could prevent fungi from affecting both hosts.

Second, the range of hosts that entomophthoralean fungi can infect has been found to be more expansive in the laboratory. This could be due to an artificially high dosage of infectious spores and stress to the host causing a weakened immune system (Keller 2002). For example, when a panel of 16 dipteran species were exposed to *E. muscae*, six species not known to acquire this infection naturally were successfully killed, though only three of these produced appreciable numbers of conidia (Steinkraus and Kramer 1987). Similarly, the entomophthoralean fungus *Entomophaga maimaiga* is naturally observed to cause epizootics just in the gypsy moth, *Lymantria dispar*, but when a panel of 78 lepidopteran species were exposed to *E. maimaiga* in the laboratory by immersion for two seconds in a 1 × 10^5^ conidia / mL solution, approximately a third were successfully infected and sporulated (Hajek et al. 1995).

**Fig. 4:**
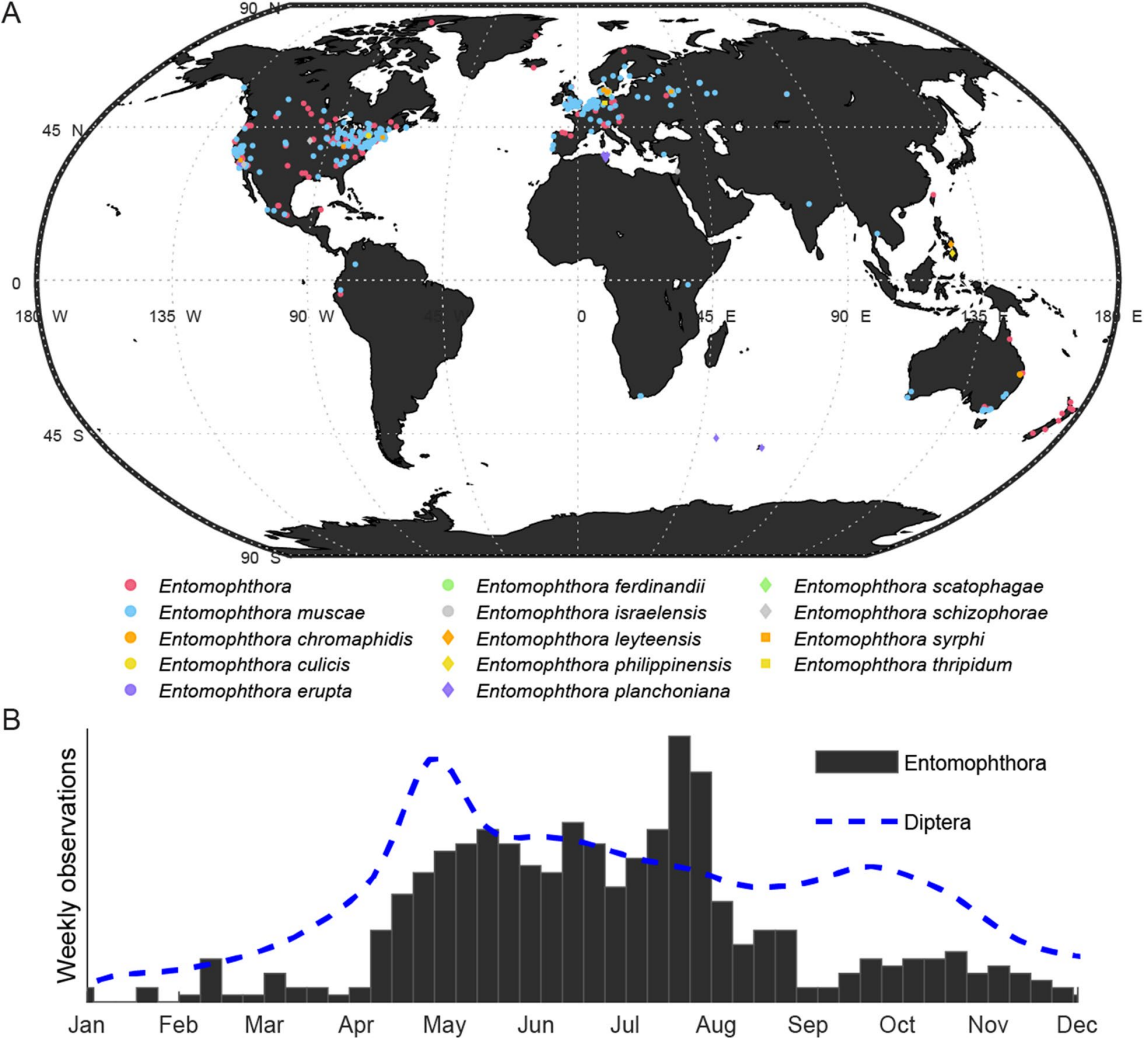
Geographical (**A**) and seasonal (**B**) distribution of recorded *Entomophthora* observations. **A** A total of 1154 observations were compiled from USDA ARSEF collection, the Global Biodiversity Information Facility (GBIF) and iNaturalist.; additional observations (geographical coordinates only) were added from Ben-Ze’ev and Zelig (1984), Villacarlos and Wilding (1994), Villacarlos et al. (2003), Ben Fekih et al. (2013), Papierok et al. (2016), and Jorgen Eilenberg (pers. comm.). Observances were only included if they listed a currently recognized *Entomophthora* species and valid latitude and longitude values. **B** Weekly frequency of observation of all *Entomophthora* species (black bars, 475 observations) overlaid with weekly frequency of observation of all dipterans (blue dotted line, 78,522 observations) based on iNaturalist data accessed on Nov, 3, 2020. Data and code (Matlab) that were used to generate this figure available are as Additional files 1 and 2 respectively

Our current understanding of the ecology and geographical distribution of *Entomophthora* is limited by relatively sparse environmental sampling compared to other studied fungal species. Only a handful of studies have sampled *Entomophthora* species systematically at a local scale (Gryganskyi et al. 2013a, b; Steenberg and Eilenberg 1995; Jensen et al. 2001), and most observations are based on sporadic sampling of usually one to very few dead fungus-infected insects from any given location. There is thus a dire need for detailed environmental sampling of most species within *Entomophthora* to determine population sizes and densities. Despite limited sampling, *Entomophthora* species appear to be broadly distributed across temperate environments and, consistent with a variety of reports, are most commonly observed in the spring and fall in the wild (Wilding 1970; Carruthers and Haynes 1986; Watson and Petersen 1993) (Fig. 4). However, *E. muscae* infections have been observed even in winter months in buildings where hosts shelter from the elements (Kramer and Steinkraus 1981; Eilenberg et al. 2013). Given what is known of the life-cycle of *Entomophthora* species (reviewed below), the broad global distribution of potential hosts for the fungus, and the fact that these fungi are woefully understudied, it seems reasonable to hypothesize that *Entomophthora* can be found throughout their host’s range, as opposed to only existing in subsets of these ranges.

In addition, it is very likely that there are several *Entomophthora* species that have yet to be discovered. For example, observations of *E. muscae*-like fungi have been made in *Coleoptera* and *Hymenoptera*, though have yet to be formally described (Eilenberg et al. 1987). First, due to lack of study and the relative obscurity of these organisms, we have effectively explored only a small fraction of *Entomophthora*-containing habitats. In addition, cadavers of insects killed by *Entomophthora* can become unrecognizable to non-experts in as little as 24 h: what is left of the host body desiccates, the remains can be consumed by saprobic fungi and/or the cadaver can be dislodged from the surface to which it is adhered. As more scientists become aware of these fungi and more of *Entomophthora*’s potential range is probed, we expect to find additional species. It is notable that six of the 21 described *Entomophthora* species were discovered within the last 20 years. Also, *Entomophthora* species are morphologically similar and as we move away from morphological-based identification of these fungi and towards sequence-based taxonomic assignment, it is likely that species designations will narrow.

## INFECTION AND THE FUNGAL LIFE-CYCLE WITHIN THE HOST

**Fig. 5:**
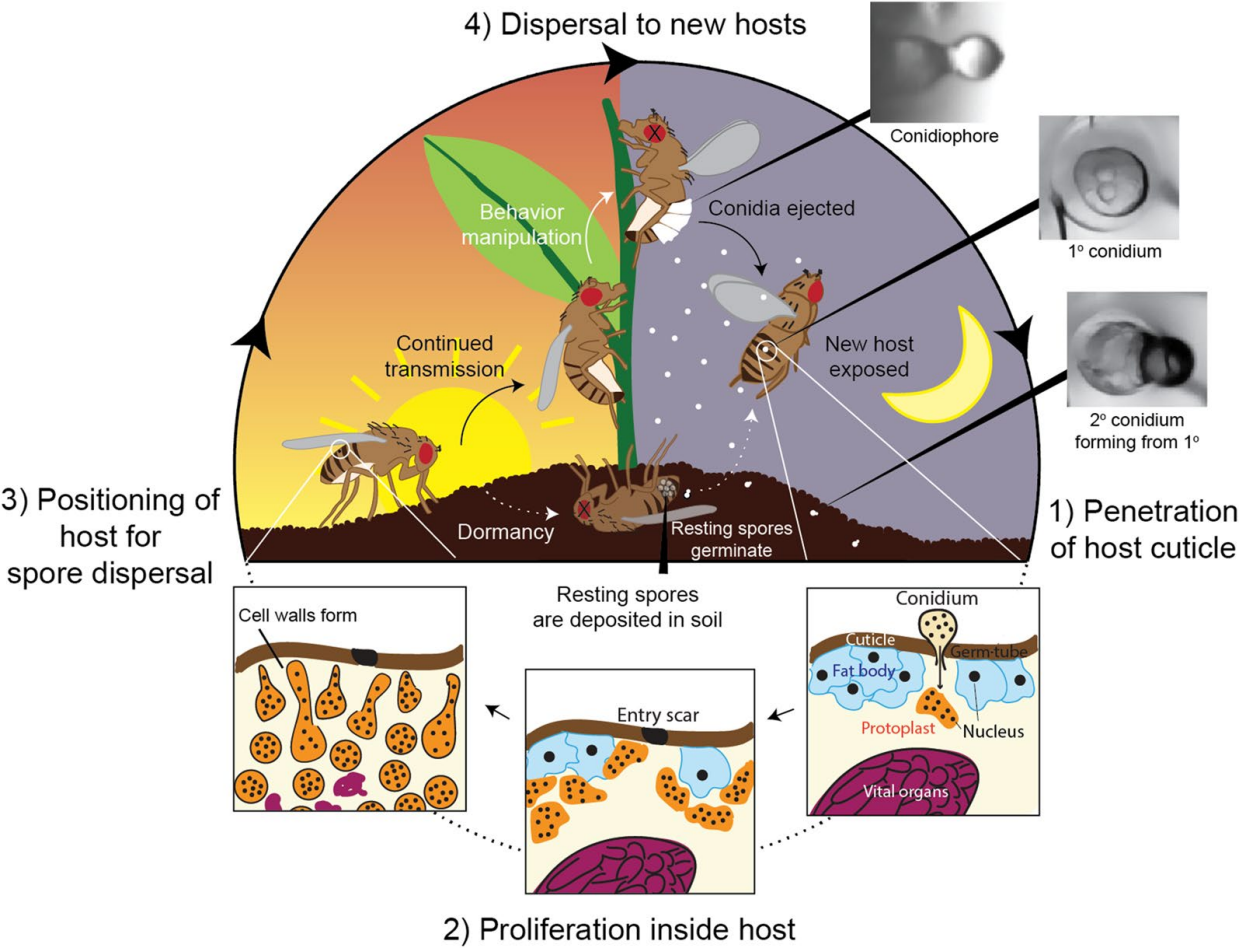
Schematic illustration of the life-cycle of *Entomophthora* fungi. The life-cycle of all *Entomophthora* species follows the same basic outline: **1** Infectious spores land on and penetrate the cuticle (right) to obtain access to the hemolymph where they assume protoplastic (i.e., cell-wall-less) morphology. **2** Protoplastic fungal cells proliferate in the host body cavity using the fat body and freely circulating nutrients as an energy source. **3** When host resources are depleted, the fungus forms a cell wall and proceeds through one of two routes: in the majority of cases, the fungus elicits a series of end-of-life behaviors (e.g., summit disease) that position the host for continued transmission (i.e., immediate infection of a new host via sporulation); alternatively, the fungus forms environmentally persistent, dormant structures (i.e., resting spores) and the host exhibits alternative moribund behavior (e.g., returning to the soil). Continued transmission (represented by the solid black line) has been observed for all species, while resting spore formation (dashed white line) has only been described for some; sporulation and formation of resting spores are mutually exclusive in a single host. **4** In the route of continued transmission, the fungus sporulates, releasing infectious conidia from spore-launching structures (conidiophores) into the environment where they can encounter new hosts; primary conidia are launched directly from the dead host, while secondary conidia form when primary conidia land on non-host substrates. Photos: C. Elya

Broadly speaking, nearly all *Entomophthora* fungi follow a common survival strategy consisting of infecting, consuming, and then behaviorally manipulating their insect hosts (Fig. 5). In summary: first, conidia launched from previously infected hosts land on the cuticle of a new host and bore through the cuticle to gain access to the hemolymph. Next, the fungus proliferates in the hemolymph using non-essential organs for food, thereby keeping the host alive. As resources dwindle, the fungus then alters the behavior of its host to position the host ideally for spore dispersal. This can occur either by forcible discharge of infectious conidia or formation of thick-walled resting spores that are capable of overwintering. Most *Entomophthora* spp. disseminate spores from host cadavers (i.e., hosts previously infected and killed by the fungus) that have become attached to elevated locations, though a handful of species (*E. erupta*, *E. thripidum,* and *E. weberi*) spread infectious conidia while their hosts are still alive (i.e., via active host transmission). Importantly, behavior modification and sporulation by *Entomophthora* fungi only occur at specific times of the day, a hallmark of *Entomophthora* biology discussed later in this section. Finally, the fungus produces and forcibly ejects conidia from the spent host to land on a new host and begin the cycle again. Here we take a detailed look at these steps of the life-cycle. As the bulk of what we understand about the course of infection for any *Entomophthora* species comes from the cadaver transmitting *E. muscae*, we base our discussion on *E. muscae*’s life-cycle, pointing out parallels and differences to other *Entomophthora* species when information is available.

### Step 1: Penetration of host cuticle

As for all *Entomophthora* species, the infection cycle for *E. muscae* begins when a conidium lands on a new host (Fig. 5—Step 1). This conidium must then germinate and penetrate the cuticle to gain access to the hemolymph. While *E. muscae* can penetrate the cuticle at any point on the body, the most common sites of landing and invasion are the abdomen. The high frequency of abdominal invasion is likely in part because the abdomen comprises the largest portion of the fly’s body, though it may also be a more favorable point of entry because it is less heavily sclerotized than other host surfaces (Brobyn and Wilding 1977). The cuticle is breached as the conidium germinates, growing a thin hyphal-like extension, termed a germ-tube, that punctures the host cuticle using both chemical (enzymatic) and mechanical force (Brobyn and Wilding 1983). The cuticle melanizes at the point of entry, though presently it is unclear if this is directly caused by the invading fungus or a response by the host’s immune system. In *E. muscae,* germination proceeds only under conditions of localized saturating humidity (Kramer 1980a, b). As a result, humidity conditions for germination dramatically impact host infection: in one study with *Delia antiqua* and *D. platura* adults, ~ 99.6% of flies died when exposed to *E. muscae* spores under saturating humidity (100%) whereas on average only 12% died when exposed under ambient humidity (65–70%), and mortality under ambient humidity varied greatly between experiments (Carruthers and Haynes 1985). The timing of germination has been observed to be quite variable for *E. muscae*, taking anywhere from two to 24 h (Brobyn and Wilding 1983).

*E. muscae*, like other *Entomophthora* spp., can only infect specific host species (Table 1), though both the basis and the precise breadth of this specificity are currently unknown. One possible point of specificity determination is recognition of host cuticle. One study of germination found that a collar formed around an *E. muscae* conidium when it landed on the cuticle of a *M. domestica* adult whereas a collar was not formed when *Conidiobolus obscurus* (another entomophthoralean fungus that is not known to infect house flies) landed on the same substrate (Brobyn and Wilding 1983). However, this same study also observed that, regardless of the formation of a collar, both fungi were able to penetrate the fly cuticle, which suggests that the cuticle is not the only barrier to establishing infection. In this vein, the entomophthoralean fungus *Entomophaga grylli* has been found to only release protoplasts from germinated conidia in the presence of host grasshopper extract, which suggests that a factor in the hemolymph is required for species specificity (MacLeod et al. 1980). This is in contrast to distantly related ascomycete entomopathogens, such as the hypocrealean *Metarhizium acridum* which requires host-specific cuticle cues to germinate and thus fails to penetrate the cuticle of a foreign host (Lovett and St Leger 2017), indicating divergent mechanisms of entomopathogenic host recognition across fungi.

*E. muscae* does not just exhibit specificity in the species of the hosts it will infect, but also in the life-stage of the host. Attempts to infect other life-stages have failed (Baird 1957, Elya, pers. obs.) and larvae and pupae have never been observed to be infected with *E. muscae* in the wild. As with host specificity, the basis for life stage specificity is also unclear. It seems likely that infection fails to occur because the conidia cannot penetrate the larval or pupal exterior, since the cuticular composition of these stages is distinct from that of adults. Still, it is also possible that some conidia do enter but fail to thrive in the absence of particular nutrients or extracellular cues.

### Step 2: Proliferation inside the host

Having gained entry into the hemolymph, *E. muscae* transitions to the next phase of its life-cycle and begins growing as protoplasts (i.e., without a cell wall) in the host hemocoel (Fig. 5—Step 2). First, the entire cytoplasmic contents of the conidium are transferred through the germ-tube into the host hemocoel to form a hyphal body (Brobyn and Wilding 1983). Once in the host hemolymph, *E. muscae* protoplasts target the fat body for consumption, using only this tissue as an energy source to proliferate and sparing all other host organs (e.g., gut, gonad, nervous system). Within the first 28 h in an infected house fly, the bulk of the proliferating hyphal bodies are located next to the heart hemocytes (Brobyn and Wilding 1983). The cells exhibit a variety of irregular shapes, which are thought to be dictated by the force of the circulating hemolymph (Brobyn and Wilding 1983). At 48 h after exposure in fruit flies, *E. muscae* cells are first consistently observed in the neuropil (the tangled mass of neuronal processes, excluding neuronal somae) of the brain and ventral nerve cord (VNC), with additional fungal cells observed in the hemolymph, usually adjacent to fat body cells (Elya et al. 2018). While fungal cells in the nervous system physically displace neuronal processes, they do not appear to actively kill, invade, or consume neurons at this stage. Invasion of the neuropil is not unique to *E. muscae*: it has been similarly observed in insects infected with other entomophthoralean fungi including *Strongwellsea castrans*, *Entomophaga grylli,* and *Conidiobolus coronatus* (formerly *E. coronata*) (Lowe and Kennel 1972; Humber 1976; Funk et al. 1993). As the infection progresses (at 72 h and 90 h after exposure, for fruit flies and house flies respectively) the hemocoel of an infected fly becomes riddled with hyphal bodies (Brobyn and Wilding 1983; Elya et al. 2018). Though fungal cells are present throughout the fly, most cells are located in the abdomen as they continue to attack the fat body and spare the fly’s vital organs.

Not all *Entomophthora* species follow the same pattern of hemocoel invasion. For example, the aphid-infecting *E. planchoniana* first concentrates most heavily in the head, rather than near the heart as observed with *E. muscae*, though both species are first observed to be most abundant near hemocytes (Brobyn and Wilding 1977). *Entomophthora erupta has been* observed to only invade the abdominal cavity of *Miridae* hosts (*Lygus communis* and *Adelphocoris lineolatus*) and not the head or thorax (Dustan 1924; Ewen 1966). That species also consumes the host gonads, thus effectively castrating the host prior to active host transmission of conidia and death. Though less detailed, descriptions of *E. thripidum* infecting host thrips suggest that *E. thripidum* is also restricted to occupying the abdomen (Samson et al. 1979). The distinct mode of host invasion (abdomen only) and tissue utilization (gonads as well as fat body) of *E. erupta* and *E. thripidum*, both active host transmitting species, may reflect a key difference in the patterns of tissue invasion and consumption patterns between *Entomophthora* species that disperse by active host transmission and cadaver transmission.

The main hypothesis as to why *Entomophthora* (and other entomophthoralean fungi) grow as protoplasts in the insect hemolymph is to aid the fungus in evading host immune recognition (Boomsma et al. 2014). Insects only have an innate immune system, meaning that instead of producing a diverse population of antibodies using somatic recombination that enable the recognition of any number of novel epitopes (termed pathogen associated molecular patterns, or PAMPs), insects can only recognize a limited repertoire of conserved PAMPs using statically-encoded pattern recognition receptors (PRRs) (Stokes et al. 2015). In insects and vertebrates alike, known immunogenic fungal PAMPs include components of the fungal cell wall (e.g., chitin, mannan, Beta-glucan) (Levitin and Whiteway 2008; Arana et al. 2009). In growing as protoplasts without the presence of cell wall residues, the fungus would lack the PAMPs that could trigger an immune response in the host. Thus, growing protoplastically could be an adaptive strategy to avoid immune recognition and conflict.

Consistent with this hypothesis, work in the generalist ascomycete entomopathogen *Beauveria bassiana* in the beet armyworm *Spodoptera exigua* has shown that in vivo produced protoplastic cells are less susceptible to phagocytosis by the insect host and recognition by a host-specific lectin (Pendland et al. 1993). A transcriptomic time course in *Drosophila melanogaster* has demonstrated a robust initial response to infection by *E. muscae* (24 h after exposure) and found an elevated immune response to persist late into infection (up to 72 h after exposure) (Elya et al. 2018). It is possible the initial immune spike occurs in response to cuticular penetration, during which cell wall components may be shed during the transition to protoplastic growth, and that the elevated response seen late into infection reflects a lingering response from this initial activation. On the other hand, it is possible that the elevated immune response observed in late-stage infection reflects a continued (albeit inefficient) recognition of fungal epitopes in the hemocoel. Clearly, further experiments need to be done to clarify the nature of the insect host’s immune response to *Entomophthora* fungi.

Unlike other entomopathogenic fungi, for example *Beauveria* (Kucera and Samsináková 1968)*,* and *Metarhizium* (Schrank and Vainstein 2010), *Entomophthora* and other entomophthoralean fungi are considered not to produce mycotoxins (i.e., poisonous substances) and instead consume all available host resources as their means of killing their host (Bidochka and Hajek 1998; Boomsma et al. 2014; Humber 1984). The absence of toxin production is hypothesized for two main reasons: (1) producing toxins would shunt metabolic resources away from fungal growth; and (2) production of toxins could lead to premature host death, killing the host before all resources are utilized or the host is optimally positioned in the environment for spore dispersal (see Fig. 5—Step 3). The assumption that toxins are not produced by *Entomophthora* should not, however, be taken for granted. In the future, this claim should be critically re-evaluated using genomic and/or proteomic data.

### Step 3: Positioning of host for spore dispersal

*Entomophthora muscae* will continue to proliferate exponentially in the host hemolymph until host resources are depleted, at which point it will need to leave the spent host and infect a new one (Keller 2002; Hansen and De Fine Licht 2017). Like many other fungi, there are two possible routes that *E. muscae* and other *Entomophthora* species can take: (1) formation and ejection of infectious conidia (i.e., sporulation) to immediately spread to a new host (Fig. 5—Step 3, continued transmission); or (2) formation of thick-walled structures called resting spores that can persist over months or years, eventually germinating to infect a new host (Fig. 5—Step 3, dormancy). Sporulation has been confirmed for all *Entomophthora* species and is the direct means of transmission to a new host. Resting spores have not yet been observed for the majority of *Entomophthora* species, though they are hypothesized to be formed across the genus (Hajek et al. 2018). This being the case, we discuss the sporulation route for the remainder of this section and address the resting spore stage later under “Survival outside of the host”.

For *E. muscae* and many other cadaver transmitting *Entomophthora* species, preparation for sporulation consists of concurrently transitioning to a new phase of growth whilst the host executes a stereotyped series of behaviors that ultimately position the fungus-filled insect for optimal spore dispersal after death (Krasnoff et al. 1995; Elya et al. 2018). The end-of-life behaviors evoked by *E. muscae* have been the subject of much fascination (Trouessart 1891; Clément 1920), not only for their consistent circadian timing but also for their uniquely dramatic presentation. Owing to the stereotypic and host specificity of these behaviors and that they appear to exclusively benefit the fungus, and not the host, these behaviors are considered to be elicited by the fungus (i.e., manipulated).

### Moribund behaviors induced by cadaver transmitting *Entomophthora*

First, flies exhibit a behavior known as “summit disease”, wherein they seek out elevated locations in their immediate environment (Evans 1989). Summiting behavior has often been inferred upon discovering *E. muscae*-killed flies (*Delia* sp*.*, *Coenosia* sp.) adhering in elevated locations in the field (e.g., clinging above the ground onto plants or fences) (Miller and Mcclanahan 1959; Berisford and Tsao 1974; Carruthers 1981; Eilenberg 1987b; Maitland 1994; Gryganskyi et al. 2013b). When end-of-life behaviors have been observed in real time, the first noticeable change in moribund *E. muscae* flies is that they cease to fly upon provocation, though it is presently unclear if lack of flying is due to physical inability (i.e., damaged musculature) or suppression of flight circuit activity (Berisford and Tsao 1974). Flies will continue to walk and climb, and, depending on substrates available in their environment, will move upwards. Eventually, elevated flies will show an unsteady gait and then stop walking altogether (Macleod 1963; Elya et al. 2018). At this point, the fly’s legs appear to spasm and their abdomen may heave up and down (Elya et al. 2018). If positioned on a narrow substrate (e.g., a plant stalk or stem), the fly may position its legs to wrap around or “hug” the substrate (Berisford and Tsao 1974).

**Fig. 6:**
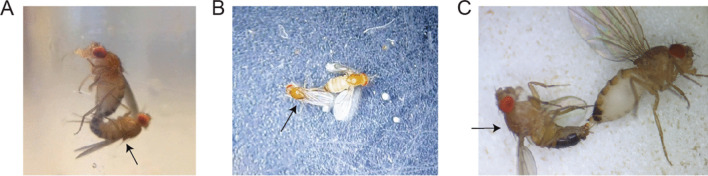
Male fruit flies adhered via genitalia to *E. muscae*-infected dead or dying flies. Each panel shows a discrete occurrence of this phenomenon. Videos of each of these occurrences can be found at https://youtu.be/R8wRNitEFuU. An arrow points to males in all panels. **A** Male stuck attempting copulation with an actively dying fly. The male’s posture is not typical of an actively copulating male (abdomen is not sufficiently curled, forelegs not being used to grip the female). The female has undergone proboscis extension but has not yet raised her wings. **B** Male engaging in grooming behavior, oriented antiparallel to the female, indicating that copulation is not actively occurring. **C** Male adhered to a dead female via genitalia, anesthetized on a carbon dioxide pad. Photos/videos: C. Elya

Next, the fly will extend its proboscis, often shakily and without opening its labellum (the labellum spreads during normal meal bouts) (Schwarz et al. 2017; Elya et al. 2018). Often, a droplet is observed to form on the proboscis tip (Berisford and Tsao 1974). If the proboscis makes contact with the surface, it will adhere, leaving the fly effectively glued in place. The nature of the adhesive material remains to be definitively determined, though it has been proposed to consist of everything from vomited food to overgrown mycelium to specialized fungal structures called rhizoids, the latter having been a major point of contention (Brobyn and Wilding 1983; Balazy 1984). On rare occasions, fruit fly males have been found copulating with dying *E. muscae*-infected fruit fly females, and these males become stuck to the dying female via their genitalia, much as dying flies become stuck to a substrate via their proboscis (Fig. 6). A parsimonious explanation for these observations is that the adhesive substance emanating from the proboscis and genitalia is the same material, and consists of fungal secretions or vegetative growth, rather than food (which exits via the anus, not the ovipositor) or specialized holdfast structures. As for the source of this material’s stickiness, a simple hypothesis is that *E. muscae* cells are coated in sticky hydrophobin proteins, which are known to be produced by many species of filamentous fungi (Linder et al. 2005).

*Entomophthora muscae*-killed flies do not always adhere to substrates via their proboscides: sometimes instead the point of attachment appears to be the legs wrapped around a substrate. Both *Entomophaga grylli* and *Entomophthora muscae* have been observed to invade the muscle tissue of their recently-dead hosts (Brobyn and Wilding 1983; Funk et al. 1993; Elya et al. 2018). In *Entomophaga grylli*, this invasion has been proposed to contribute to immobilizing the cadaver in situ; this may also be the case for *E. muscae* and its fly hosts (Funk et al. 1993). Given the experimental evidence in *Erynia neoaphidis* that elevated hosts are able to spread spores over a wider area (Hemmati et al. 2001) as well as the repeated appearance of summiting behavior not just in *Entomophthora* and other *Entomophthorales* but more broadly across fungi (e.g., some *Ophiocordyceps* spp. (Andersen et al. 2009), viruses (e.g., baculovirus; Hoover et al. 2011), and helminths (e.g., *Dicrocoelium*; Carney 1969)), elevating hosts probably confers an important enough dispersal advantage to favor evolving redundant mechanisms to maintain host elevation.

Finally, up to two hours after the proboscis has been extended, the wings of the *E. muscae*-infected dying fly will raise up and away from the dorsal abdomen (Krasnoff et al. 1995). The wings raise up quickly, usually only taking 15 min (Elya et al. 2018; Krasnoff et al. 1995). This behavior provides a clear advantage to spore dispersal: most spores are ejected from the fly’s dorsal abdomen, which is covered by its folded wings while the animal is not in flight. Moving the wings away from the dorsal abdomen provides a clear path for launched spores into the surrounding environment. Raised wings have also been reported in phorid and sciarid flies killed by *E. culicis*, gall midges killed by *E. israeliensis*, simuliids killed by *E. simulii,* and syrphids killed by *E. syrphi* (Gol’berg 1979; Ben-Ze’ev and Zelig 1984; Keller 2002). The posture of the wings can vary between different hosts: while *M. domestica* and *D. melanogaster* raise their wings to almost perpendicular to the body axis, yellow dungflies (*Scatophaga stercoriaria*) infected by *E. scatophagae* (a member of the *E. muscae* species complex) raise their wings out rather than up (Maitland 1994). *Delia kullensis* infected by *E. ferdinandii* (another member of the *E. muscae* species complex) also displays spread rather than lifted wings in its death pose (Keller 2002). Distinct wing positioning has also been observed in insects killed by other entomophthoraleans: soldier beetles and goldenrod beetles killed by *Erynia lampridarum* both fold their wings back upon death (Carner 1980; Steinkraus et al. 2017).

The mechanistic bases for fungal-induced summiting, proboscis extension, or wing-raising manipulated behaviors, are not understood (see Lovett et al. 2020b for a recently posed hypotheses that summiting might be related to insect sleep behavior). While all could potentially be due to neuronal manipulations, fungal-induced proboscis extension and wing-raising could arise solely due to mechanical force. (This is distinct from fungal-induced summiting, which, due to its complexity, is highly unlikely to be explained by mechanical force alone.) When a fly is injected full of liquid, it will bloat, leading to the extension of its proboscis by steric exclusion (Krasnoff et al. 1995). Infected flies become very bloated as they become filled with *E. muscae* cells, and some in the field have proposed this bloating leads to proboscis extension (Brobyn and Wilding 1983). Somewhat analogously, wing-raising could be caused by the fungus physically impinging on wing muscles causing them to contract, as has been suggested for *Erynia lampridarum*-infected goldenrod beetles (Steinkraus et al. 2017).

#### Fungal morphology in the moribund host

While host behavior is being manipulated, *E. muscae*’s cellular morphology is changing inside the fly, though the precise timing of the morphological transition with respect to behavior manipulation has not been definitively resolved. At least as early as the point of flight cessation in fruit flies, *E. muscae* cells within the body cavity have adopted a consistent spherical morphology (Elya, pers. obs.), which is likely achieved by forming a cell wall that gives hyphal bodies structure they were previously lacking. Similarly, *E. muscae* in house flies have been noted to shift from protoplast growth to more elongated hyphal threads *ca*. 10 h before death (Jensen 2001). The first walled cells are observed to grow hyphal-like extensions towards the host cuticle, making individual *E. muscae* cells appear as tadpole-like entities as they differentiate into conidiophores (Berisford and Tsao 1974; Brobyn and Wilding 1977, 1983). Conidiophores will not penetrate out through the host cuticle until after death. By the time conidiophores first emerge, the gut and gonads are usually destroyed, the nervous system has begun to be degraded, and the thoracic musculature is still largely intact (Brobyn and Wilding 1983; Elya et al. 2018).

#### Circadian timing of moribund behaviors

Critically, death by *E. muscae* and the morphological and behavior changes that directly precede it always occurs during a specific circadian window, with most hosts expiring four or so hours prior to sunset (Krasnoff et al. 1995; Elya et al. 2018). Even if a late-stage infected host (i.e., a host with very little fat body remaining) survives past sunset on a given day, it will not undergo stereotypical behaviors, death and sporulation until sunset the following day (Elya pers. obs.; De Fine Licht, pers. obs.). This specific timing is thought to be adaptive for the fungus, ensuring the best possible environmental conditions for sporulation and germination, a topic we explore in the next section. Specific circadian timing of moribund behaviors and death has also been observed in *E. planchoniana* and active host transmitting *E. erupta*, as well as other entomophthoralean species, *Erynia neoaphidis* and *Entomophaga grylli,* suggesting this is likely a common feature of infection by *Entomophthora* species, if not broadly among infection by entomophthoralean fungi (Dustan 1924; Pickford and Riegert 1964; Milner et al. 1984).

Given the prevalence of timed death throughout *Entomophthora* and in other *Entomophthorales*, it seems more likely that the circadian control of host death is controlled by *Entomophthora* rather than dictated by each different host species. From an adaptation perspective, timing host death and subsequent emergence to coincide with favorable humidity and temperature has clear potential implications for fungal fitness (though, importantly, the impact of circadian timing of death on fungal fitness has not been explicitly tested), while any potential benefit to the host is unclear. Indeed, available evidence so far favors the hypothesis that the fungus determines the stereotyped timing of behavior manipulation and death. When house flies entrained on a light:dark cycle were exposed to *E. muscae* and incubated in complete darkness, flies died of *E. muscae* infection randomly throughout the day (Krasnoff et al. 1995). However, when flies were exposed to *E. muscae* and held for three days on a light:dark cycle before transferring to complete darkness, flies died from fungal infection with an approximately circadian periodicity. Both flies and fungi are known to have molecular circadian clocks, networks of genes whose expression oscillates consistently over a period of about 24 h (Dunlap and Loros 2017). These clocks enable organisms to keep time in the absence of environmental cues like light or temperature. The aforementioned study demonstrated that the host clock is not sufficient to drive circadian timing of death by *E. muscae*, and suggests that an alternative mechanism (perhaps a fungal clock that requires entrainment during the protoplastic stage of growth) drives this phenomenon.

#### Active host transmission

Active host transmitting *Entomophthora* species also elicit host behavioral changes that serve to enhance spore dispersal, though spore dispersal occurs while the hosts are still living. We know far less about active host transmitting than cadaver transmitting *Entomophthora* species, with the bulk of our understanding coming from work on *E. erupta*. As previously mentioned, *E. erupta* is selective in its invasion of the mirid hemolymph, restricting itself to the abdomen where it destroys the gonads and fat bodies, and leaves the thorax and legs intact (Dustan 1924; Ewen 1966; Ben-Ze’ev' et al. 1985). This selective invasion is thought to be key for keeping the host mobile during spore dissemination. Once the abdomen is completely filled with *E. erupta* hyphal bodies, these cells differentiate into club-shaped conidiophores leading to the rupture of the abdominal cuticle, usually down the dorsal line, to reveal a continuous layer of these conidiophores (Dustan 1924). Analogous to the consistent timing of behavioral manipulation and death by *E. muscae*, this rupturing of mirids by *E. erupta* consistently occurs at a particular time of day: late at night or in the very early morning (Dustan 1924). The timing of conidiophore formation is such that spores will begin to be launched while the morning dew is still present, which likely serves to provide optimal conditions for both sporulation and germination.

Infected, abdominally-ruptured mirids continue to be active without apparent ambulatory defects, allowing them to disperse spores over a broader range than if they were incapacitated (Dustan 1924; Ben-Ze’ev' et al. 1985). Healthy mirids have been observed to feed on the conidiophore-filled abscess, placing them in close proximity to firing spores. Mirids with external signs of fungus are surprisingly long-lived, most die one to two days after abdominal rupture though some have been observed to live up to a week after rupture (Ewen 1966). One study looking specifically at neuroendocrine centers (the neurosecretory A and B cells), observed cessation of that neurosecretory material accumulation in A cells three days after infection (Ewen 1966). This was coincident with hypertrophy of the corpora allata (CA), a conserved neurohemal organ in insects (Ewen 1966). The authors could not conclude if the enlargement of the CA was a result of parasitic castration (eliminating feedback from the ovaries has been shown to lead to CA hypertrophy in several insect species (Ewen 1966)), or some other process. Regardless, that a hormonal release center is altered during infection may provide future clues as to the mechanistic basis of host behavioral changes in this system.

Active host transmission is a strategy used in other *Entomophthorales*, notably *Massospora cicadina* and *Strongwellsea castrans*. Cicadas infected with *M. cicadina* will eventually lose part of their abdominal segments revealing a white-colored fungal plug that consist of conidiophores which release spores while the cicada continues to move around (Boyce et al. 2019). It was recently revealed that *M. cicadina* releases psychoactive chemicals during infection, which are speculated to contribute to keeping the insect alive despite missing half of the body by increasing insect sexual behaviors and reducing insect feeding behaviors (Boyce et al. 2019). That the highly host-specific entomophthoralean fungi may manipulate insect sexual behaviors would seem to be an ideal way of ensuring conspecific contact between susceptible hosts, but does not imply that these fungi can be considered as sexually transmitted diseases (Hansen and De Fine Licht 2019). In general, active host transmission is well known from a number of fungal pathogens (Lovett et al. 2020a), but is not the norm and can to some extent be considered as the pinnacle of host-specific adaptation because of the intricate fungal machinery likely required to keep the host alive during fungal sporulation.

### Step 4: Dispersal to new hosts

*Entomphthora muscae* and other cadaver transmitting *Entomophthora* species seek a new host immediately after the previous one has been killed. Under laboratory conditions, *E. muscae* infected fruit flies usually die from fungal infection four or five days after exposure (Elya et al. 2018); house flies die five to seven days after exposure (Kramer and Steinkraus 1981; Hansen and De Fine Licht 2017). Time from exposure until death from *Entomophthora* species can range from two to twelve days (Macleod 1963), and has been observed to vary with several factors including incubation temperature (Carruthers and Haynes 1985; Eilenberg 1987a), spore dosage (Bellini et al. 1992), host species (Steinkraus and Kramer 1987), and body size (Mullens 1985).

#### Formation and dispersal of conidia

After the death of the old host, *E. muscae* conidiophores begin to pierce through the weakest points of the fly’s cuticle, usually the intersegmental membranes, sometimes the ventral abdomen and rarely the neck (Fig. 5—Step 4). Conidiophores arise from cell-walled hyphal bodies that project hyphal-like projections that extend towards the host cuticle. These finger-like structures emerge through the cuticle within a few hours after the host has died, first appearing as blunt outgrowths that then narrow to a partially opened septum at the tip (Mravec et al. 2014). A single conidium forms at the top of each conidiophore by the transfer of most or all conidiophore nuclei along with cytoplasm through the opened septum (Keller 2002). Once mature, the septum completely closes, and cytoplasm continues to build pressure behind the closed passageway. Eventually, enough pressure accumulates that the conidium is violently ejected into the environment, traveling at an initial velocity of 10 m/s (Elya et al. 2018).

Though there was once disagreement regarding the ejection mechanism of *E. muscae* primary conidia from conidiophores, recent work has conclusively demonstrated that primary conidia are fired using a water cannon mechanism (de Ruiter et al. 2019). Each primary conidium is surrounded by a characteristic “halo” of material when landed on a surface. Based on microscopic analysis of landed spores, the source of this halo was proposed to be either co-ejected cytoplasm (assuming a water cannon mechanism of spore launch) (Humber 1981) or a product of membrane rupture as the spore came into violent contact with the surface (Eilenberg et al. 1986). High-speed video clearly demonstrated that the halo lands concurrently with the primary *E. muscae* conidium, indicating that it is co-ejected (Elya et al. 2018). Additional work using a biomimetic water cannon system was able to accurately model primary spore launch (de Ruiter et al. 2019). Interestingly, this work found that *E. muscae* conidia fall within the predicted size regime of projectiles which can be successfully ejected in this model, large enough to counteract aerodynamic drag and move away from the fly, and small enough to be launched with substantial velocity (de Ruiter et al. 2019). It is likely that the water cannon mechanism applies to the launching of primary conidia in all *Entomophthora* species, though similar work has not yet been completed for these fungi. While arguing over the source of a gooey halo may seem trivial, the halo surrounding the primary conidia is not merely a decorative by-product of spore launch. Removing the halo via dissolving it in water has been found to prevent further growth, suggesting that the halo is necessary for the normal life-cycle progression of *E. muscae* (Baird 1957). Other putative functions for the halo include protecting the spore inside upon hard contact with the surface as it lands, providing a source of adhesion to the surface it lands upon and keeping the conidium hydrated so it is competent to generate secondary conidia (Humber 2016).

For *E. muscae* infected flies, the first primary conidia are ejected around four to five hours post-mortem and continue to fire for the next 18–20 h under ambient conditions (Mullens and Rodriguez 1985; Elya et al. 2018). While primary conidia fire autonomously over this time period, they can also be triggered to launch v*i*a mechanical stimulation, for example by a curious fly inspecting a cadaver (de Ruiter et al. 2019). Ejecting spores in response to mechanical stimulation likely provides an additional dispersion advantage, ensuring that spores are launched if and when a host comes into contact with the cadaver.

If a primary conidium does not land on a susceptible insect host, it will typically sporulate once again to form a smaller, secondary conidium (Macleod 1963). Secondary conidia arise by budding off from primary conidia. *E. muscae* secondary conidia can start to form from primary conidia as soon as they land (Humber 2016). The cytoplasm of the primary conidium is transferred to the secondary, leaving behind an empty primary conidium, termed a ghost. In contrast to primary conidia, secondary conidia are fired by papillar eversion (Humber 2016), a process reminiscent of the sudden flipping of a child’s rubber popper. Most secondary conidia launch around 4 h after primary discharge, but can eject a new conidium as late as 9–10 h after primary discharge (Mullens and Rodriguez 1985). If a secondary conidium fails to find a host, it can sporulate again to give rise to a tertiary conidium, provided there is adequate energy and hydration available for this process (Macleod 1963). While formation of higher order conidia has been observed (i.e., tertiary and beyond), it is not typical for these fungi to form them (Mullens and Rodriguez 1985).

#### Germination: completing the life-cycle

Once on the host cuticle, the conidium must next germinate to form a germ-tube that penetrates through the cuticle and provides access to the hemolymph. The fungus thus returns to the beginning of its life-cycle (Fig. 5—Step 1). Like host death, conidiophore formation and sporulation, germination is also time-sensitive. Under ambient conditions, conidia quickly lose their ability to germinate: while some have observed germination after two weeks, a more typical time window is approximately 24 h (Macleod 1963; Madeira 1998; Kalsbeek et al. 2001b). There is currently a lack of consensus when it comes to which type of spore (primary or secondary) is responsible for germinating and bringing the cycle of infection full circle. While some state that viable primary conidia always form secondary conidia, even if they land on a susceptible host (e.g., Güssow 1917), other studies have reported the formation of secondary conidia *only* in instances where the primaries failed to land on the host (Thaxter 1888; Burger and Swain 1918; Steinhaus 1949) or noted failure to observe successive generations of conidia forming on a host cuticle (Brobyn and Wilding 1983). From a purely metabolic perspective, the latter scenario (secondaries only form when primaries fail) makes much more *a priori* sense than the absolute requirement to form secondaries regardless of substrate. Forming a secondary conidium from a primary that is already landed on a host takes precious time and energy, not to mention that this secondary may be launched off the host cuticle and therefore further from the host. That said, it is possible secondary conidia are uniquely equipped for either host recognition or germination, or that the timing of secondary formation and firing is aligned with host activity, so they must be formed regardless of circumstance. Studies reporting that germ-tubes are formed either exclusively (Kramer 1980a, b) or predominantly from (Carruthers et al. 1985) secondary conidia and that secondary conidia are more infectious than primary conidia (Bellini et al. 1992) support this possibility. As clarifying both the growth and infection competencies has implications for understanding *E. muscae* biology more broadly, these questions are in dire need of further investigation.

#### Abiotic factors affecting spore dispersal and germination

Much attention in the *E. muscae* literature has been given to the role that environmental conditions, especially humidity, play in sporulation for both primary and secondary conidia, germination, and infectivity (Table 2). While several authors have concluded that higher humidity leads to better sporulation (Kramer 1980a, b), others have reported that sporulation can occur over a range of relative humidity (Mullens et al. 1987; Watson and Peterson 1993; Madeira 1998). The default assumption that high humidity is required for optimal sporulation, therefore, is probably not accurate. The consensus for humidity requirements for germination, however, is more straightforward. Germination has been consistently reported to occur more efficiently or exclusively under conditions of high, usually saturating, humidity (Kramer 1980a, b; Carruthers and Haynes 1986). Infectivity, like sporulation, fluctuates with humidity but can occur in both dry and wet conditions (Kramer 1980a, b; Madeira 1998). Since successful host infection necessitates both sporulation and germination, the understanding that sporulation can occur under a range of humidity conditions while germination must proceed with high humidity may initially seem paradoxical. If this is true, how can hosts be infected under low humidity? A proposed explanation as to why infection can persist in dry conditions is that the boundary layer surrounding the fly cuticle is at the saturation point, so is amenable to germination (Kramer 1980b).

**Table 2:** Reported ideal conditions for *E. muscae* across life-stages

Factor	Sporulation	Germination	Host infectivity
Temperature	~ 20 °C (Watson and Peterson 1993; Kalsbeek et al. 2001a, b)	21 °C (Carruthers and Haynes 1986)	21 °C (Madeira 1998)
Humidity	20–100% (Mullens et al. 1987; Watson and Peterson 1993; Madeira 1998)	Saturating humidity (Kramer 1980a, b; Carruthers and Haynes 1986)	Saturating humidity (Carruthers and Haynes 1985)
Host age	N/A	N/A	Young (*Drosophila*: 0–6 d post-eclosion) (Elya et al. 2018; Mullens 1985)
Host genotype	N/A	N/A	Unknown host genetic factors (Wang et al. 2020)
Host density	N/A	N/A	High (Carruthers et al. 1985)

N/A—no data available

The role of temperature in *E. muscae*’s life-cycle has been examined in several studies (Table 2). In house flies, strains of *E. muscae* have been observed to produce primary conidia from 7 to 38 °C, with peak conidial production being reported anywhere from 7 to 20 °C (Watson and Peterson 1993; Kalsbeek et al. 2001b). Lowering the temperature extends the duration over which conidia are released, extending the window of release from about 24 h at 21 °C up to 120 h at 7 °C (Watson and Peterson 1993; Kalsbeek et al. 2001b). Secondary conidia formation and germination has been observed at temperatures ranging from ~ 4 to  ~ 27 °C (Carruthers and Haynes 1986) though optimal infectivity and germination have both been reported to occur at 21 °C (Carruthers and Haynes 1986; Madeira 1998). Time from exposure to death decreases with increasing temperature: *Psila rosae* exposed at ~ 27 °C died by 4 d after exposure to *E. schizophorae*, while hosts exposed at 5 °C were still succumbing to fungal infection 39 d after exposure (Eilenberg 1987a).

Despite the attention paid to humidity and temperature to infectivity of *E. muscae*, evidence suggests that these factors are not the most critical in determining natural infection spread and resultant mortality in the wild. Regression analysis on multiple environmental factors, including temperature and humidity, found that host density (the number of hosts in a given volume of space) and inoculum density (the number of spores landed on a host) were the only significant variables that correlated with infection outcome (Carruthers et al. 1985). Anecdotally, each of us have independently observed that the likelihood of encountering *E. muscae* in the wild has been consistently correlated with a large number of hosts in the same place at the same time (Elya, pers. obs.; De Fine Licht, pers. obs.). That said, host abundance fluctuates seasonally, changing in response to environmental conditions, so while temperature and humidity may not be the most defining factors for fungal spread, they still are clearly important.

#### Biotic factors that govern infectivity

Laboratory-based studies have found that infectivity also varies with host age (Table 2). In fruit flies, *E. muscae* consistently infects and kills younger flies (within 6 d post-eclosion) at a higher rate than older individuals (Elya et al. 2018). In house flies, younger flies have also been observed to significantly exceed older flies in their rate of death connected to sporulation after exposure to *E. muscae* (Mullens 1985). The older flies tend to have a higher overall mortality rate but a lower rate of "productive" infection, i.e., infection leading to fungal dispersal. One hypothesis as to why younger hosts are more susceptible to productive *E. muscae* infection is that their cuticle is easier to penetrate. Flies that have just emerged from the pupal case have a soft, pliable cuticle that begins to harden over the next few hours due to the actions of the neuropeptide bursicon (Fraenkel and Hsiao 1965). Even after the initial tanning is completed, flies continue to secrete and deposit layers of cuticle daily in a circadian fashion (Ito et al. 2008). Thus, the cuticle grows thicker over the fly’s lifetime. The harder cuticle of older flies may impede penetration by germinating conidia, making older flies more challenging to infect.

In addition, fly susceptibility also varies with host genotype (Table 2). A recent study using a panel of inbred wild-type *D. melanogaster* observed a broad range of fly susceptibility to *E. muscae*, ranging from 1.6% to 94% mortality at the extremes (Wang et al. 2020). Interestingly, the pattern of susceptibility to *E. muscae* showed both common trends with respect to susceptibility to the generalist fungal entomopathogen *Metarhizium robertsii* and opportunistic bacterial pathogen *Pseudomonas aeruginosa*, suggesting shared mechanisms for pathogen resistance, as well as divergences, reflecting the specificity of the *E. muscae*-*Drosophila* interaction. While females were on average slightly more susceptible than males, this trend was not significant and has also been found to not be significant in studies with *E. muscae*-infected house flies (Mullens 1985).

### Other host behavior alterations elicited by *Entomophthora*

While summiting, proboscis extension, and wing raising are the most commonly described manipulated behaviors in *E. muscae*-infected flies, additional behavioral differences have also been reported in infected versus healthy hosts. However, it is important to keep in mind that just because a behavioral difference is observed in a host infected with *Entomophthora* (relative to an uninfected host), this alone does not indicate that the behavior is being elicited by the fungus, and, even if it is, that the elicited behavior is adaptive for the fungus. Manipulated behaviors are differentiated from behaviors that change in response to the infection by benefiting fungal fitness more than host fitness (often, they are exclusively beneficial to the fungus) and uniquely elicited in response to infection by a given fungus (i.e., not a general result of sickness or malnutrition).

First, flies infected with *E. muscae* that form resting spores rather than conidia have been reported in soil, rather than in elevated locations, and are not observed to adopt the stereotyped death pose of cadavers that will sporulate (Carruthers et al. 1985). The common interpretation is that the fungus either does not elicit any behavioral manipulation or elicits an alternative behavioral program in these flies, directing them to move towards the ground so that the spores inside can be deposited in the soil. Though this phenomenon, which we term “grounding behavior”, has not been heavily observed or documented for *Entomophthora*-infected hosts, the interpretation that it is a manipulated behavior is supported by several similar observations in other entomophthoralean fungi-host systems (Hajek et al. 2018).

Carrot flies infected by *E. schizophorae*^3^ have been observed to lay fewer eggs than their uninfected counterparts (Eilenberg 1987b). Since egg production requires substantial resources, decreased egg laying may reflect that infected flies simply do not have enough nutrients to produce as many eggs as uninfected flies. There is a well-documented trade-off between fecundity and immune response in animals: reduced fecundity is commonly-observed in sick animals (Tompkins and Begon 1999). Infected carrot flies were also noted to deposit eggs in aberrant locations, either away from carrot plants kept in cups (in the laboratory) or atop carrot leaves (in the wild) (Eilenberg 1987b). These observations are likely explained by the known decrease in activity of late-stage infected flies (Elya et al. 2018) as well as elevation seeking (summit disease) in flies hours before death. It is also unclear how this aberrant behavior would benefit either the fungus or the host, supporting the idea that it is not manipulated by the fungus; more work needs to be done to confirm this hypothesis.

Infected house flies have been observed to show distinct thermal preferences in response to *E. muscae* infection. When infected flies were allowed to freely explore a thermal gradient, flies in early stages of disease progression preferred temperatures warmer than those preferred by controls, while infected flies in later stages preferred cooler temperatures (Watson et al. 1993). On a dairy and swine farm, experimentally- and naturally-infected flies in early stages of infection were more likely to be found perched on warm substrates (heat lamps or sun-bathed areas) than on cool substrates 0–2 d before death (Kalsbeek et al. 2001a). In ectotherms, the altered preference for warm substrates in response to infection is a commonly observed phenomenon, “behavioral fever” (Louis et al. 1986). Using this behavior, infected animals can effectively increase their body temperature to impede pathogen growth and boost immunity (Ouedraogo et al. 2003; Wojda 2017), thereby increasing their chances of clearing the infection or postponing their demise. In the aforementioned studies, house flies that were allowed to behaviorally fever substantially reduced their mortality rates, with just 3% of flies that fevered 48 h after exposure succumbing to infection (Watson et al. 1993). Similarly, flies forcibly subjected to high temperatures (40 °C) for as little as 1 h had reduced mortality compared to flies held at room temperature, and this incubation also correlated with an increase in time until death, i.e., delayed mortality (Watson et al. 1993). House flies demonstrate behavioral fever in response to various non-*E. muscae* pathogens, so the early-infection thermal preference should be interpreted as a general pathogen response rather than an *E. muscae*-specific behavior.

On the other hand, end-of-life cold-seeking could be a result of fungal manipulation. Cold-seeking behavior has also been observed in fruit flies exposed to the generalist fungal entomopathogen *Metarhizium robertsii*, but, like behavioral fever, cold-seeking in this instance appears to reflect a more general pathogen response as it appears within the first 24 h after exposure and can be elicited by either live or heat-killed fungus (Hunt et al. 2015). *Entomophthora muscae* optimally spreads to new hosts under cool, humid conditions, so placing a host in such conditions would provide a clear benefit to the growing fungus (Carruthers and Haynes 1986). Clearly, additional work is needed to determine if cold-seeking in *E. muscae*-infected flies represents a fungal-manipulated behavior or a host response to slow disease progression.

Strangely, uninfected house flies have been reported to show enhanced sexual attraction to *E. muscae*-killed cadavers. When given a choice between an uninfected dead female and a female freshly killed by *E. muscae*, male house flies were quicker to explore and make sexual advances towards the latter (Moller 1993). This phenomenon has been interpreted as another manipulative effort by the fungus to promote its own spread, by chemical and/or visual cues. A subsequent study found that this attractiveness was not attributable to increased sex pheromones, finding that *E. muscae*-killed flies had lower levels of pheromones than their control counterparts, suggesting that other volatile compounds or the visual appeal of a swollen female abdomen were responsible for eliciting male arousal (Zurek et al. 2002). Interestingly, male attempts at copulating with infected female cadavers has so far not been observed in the laboratory-based fruit fly system (Elya, pers. obs.), but the characteristic mating-dance and wing-flicking of healthy fruit fly males has been observed towards female fruit fly cadavers infected with *E. muscae* isolates from house flies (De Fine Licht, pers. obs.). This could reflect the more aggressive and promiscuous mating tendences of house flies (house flies will try to mate with uninfected cadavers, while fruit flies do not) or strain-specific differences in sexual attraction between *E. muscae* isolates.

### Survival outside the host

As *Entomophthora* fungi are obligate pathogens and only proliferate on or inside the host body, life-stages outside of hosts are all related to transmission. As mentioned above, resting spores have been observed for some *Entomophthora* species, though they are thought to likely be formed by all of these fungi. These are thick-walled fungal spores more resistant to environmental stress. Resting spores are considered to typically be formed in older hosts in response to changing environmental conditions (decreasing photoperiod and/or temperature), and as such resting spores help *Entomophthora* species to withstand adverse environmental conditions (MacLeod 1956; Hall and Halfhill 1959). Almost a century ago, Goldstein (1923) reported thick-walled spores in desiccated *Empusa muscae-*killed corpses, positing that resting spores had been rarely observed up until his study because researchers had not been looking in old enough specimens. He proposed that resting spores formed from remaining hyphal fragments under dry conditions when conidial discharge was no longer possible, thus suggesting that resting spores could form inside an individual that had already discharged conidia. Eighty years ago, Petrishcheva, as cited in Brumpt (1941) reported nearly 100% infection of flies being treated with one year old pulverized dead fly cadavers, which likely contained resting spores.

More recently, the thinking has been that a single individual will either produce conidia or die with resting spores inside, but usually not both at the same time (Hajek et al. 2018). Carruthers et al. (1985) reported that the behavior of infected flies with resting spores was different from flies that would die via the “normal” summit-disease, providing evidence that the entire infection cycle is altered in preparation to form resting spores. Instead of summiting, hosts that formed resting spores were found to have died on the soil, with their bodies eventually dissolving to release the spores inside. That *E. muscae* lies dormant in the soil was suggested since fly pupae collected with soil became infected 10–33% of the time, whereas pupae collected and placed in sterile soil never became infected (Carruthers et al. 1985). Under favorable environmental conditions (including increased photoperiod), resting spores can germinate and produce germ conidia (i.e., turn into infectious propagules) (Macleod 1963; Tyrrell and MacLeod 1975). This suggests that *E. muscae* resting spores infect fly pupae in the soil, or the adult fly gets infected from resting spores on the outer surfaces of the pupae as it emerges, thereby providing an alternative infection route (see Fig. 5).

In temperate regions where the host insect disappears during the winter season, resting spores are considered to be the overwintering stage of the fungus. That resting spores are seasonally or ecologically driven is also supported by resting spores never having been observed in *E. muscae* isolates from house flies caught in artificially-heated cowstables in Denmark (Thomsen et al. 2001). Although fly populations oscillate inside farm buildings, a few house flies are always present so it is thought that *E. muscae* can slowly spread via conidia from host-to-host during winter. This is similar to the slow disease transmission of *E. schizophorae* between hibernating *Pollenia* spp. fly hosts that overwinter in clusters in heated attics (Eilenberg et al. 2013). In contrast, *E. muscae* isolated from cabbage flies (*Delia radicum*) in Denmark are more prone to form resting spores (Thomsen and Eilenberg 2000; Thomsen et al. 2001). Resting spores have also been observed in vitro (Eilenberg et al. 1990), but exact clues to what triggers their formation is not clear.

**Fig. 7:**
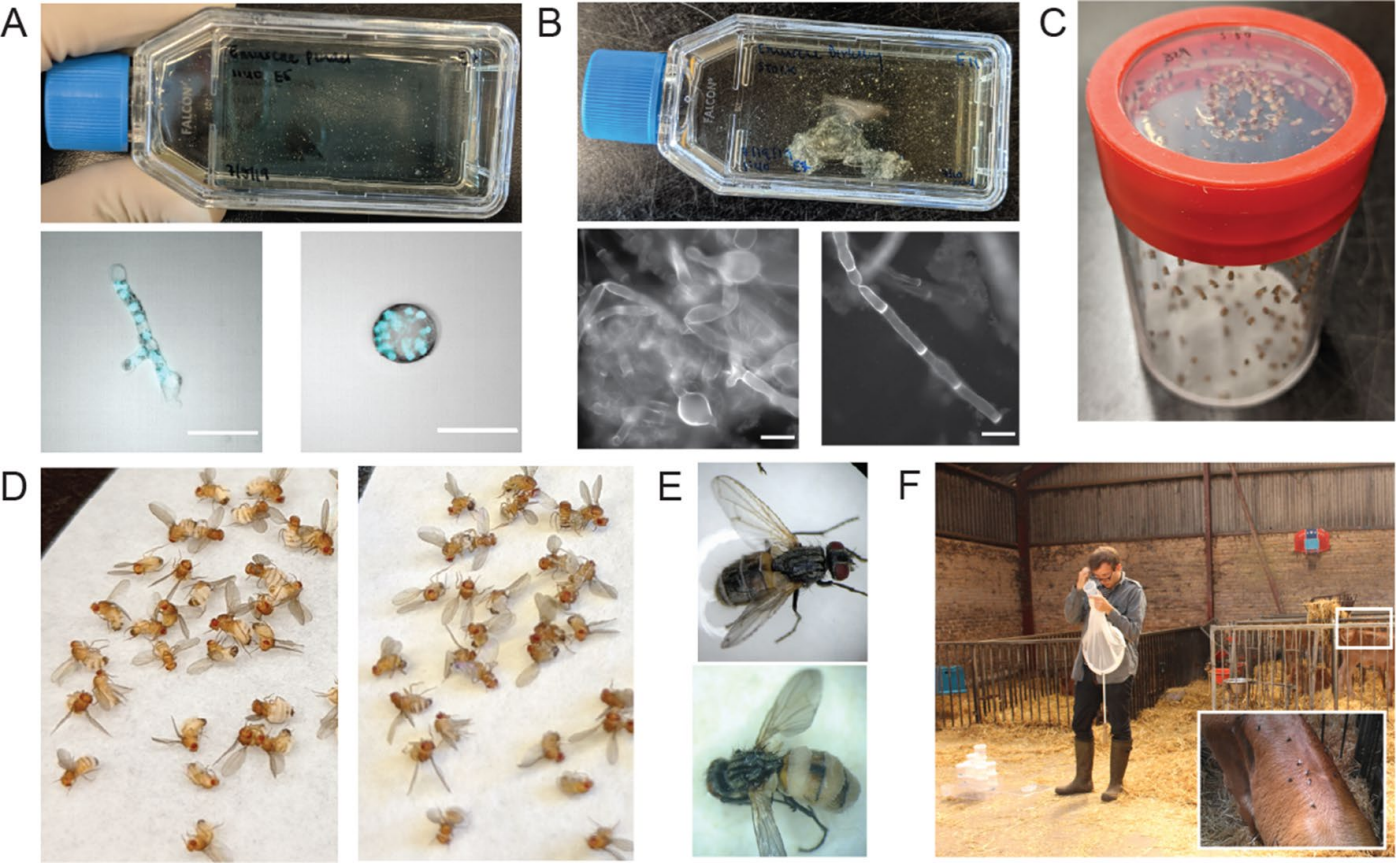
Working with *E. muscae*. **A** Top: “Young” in vitro* E. muscae* culture (72 h after inoculation); small white clusters of cells are hyphal bodies with varying morphologies. Bottom: example cell morphologies observed in vitro, stained with Hoechst 33342 to label nuclei. Bars = 5 µm. **B** Top: “Old” in vitro* E. muscae* culture (approx. one month after inoculation); the large clump of material consists of mycelial (cell-walled) tissue. Bottom: images of mycelial in vitro growth, color from staining with Calcofluor. Bars = 5 µm. **C** Hundreds of fruit flies exposed to *E. muscae *via fresh, sporulating cadavers in a small embryo collection cage. **D** Example cadavers collected from in vivo propagation of *E. muscae*. Left: cadavers collected on day of death; Right: cadavers 24 h after collection. **E** House flies killed from *E. muscae* infection from abdominal injection of in vitro culture; flies were collected at similar times of day (both between 3–4 h after sunset) but showed variability in extent of conidiophore formation. Flies died 15- and 13 d following injection, for top and bottom images respectively. **F** Collecting *E. muscae*-infected flies in the field (cow stable in Denmark); H. H. De Fine Licht is pictured scrutinizing captured *Musca domestica* flies for signs of fungal infection. Inset: *Musca domestica* flies sitting on the back of a cow in the stable. Photos: C. Elya and H. H. De Fine Licht

Part of the explanation for why there are so many unanswered questions of the basic biology of *Entomophthora* comes from the difficulty of growing many *Entomophthora* species in vitro in the laboratory (Fig. 7). While not impossible, it is not easy, and the fungal cultures may quickly change morphology and phenotypic traits during successive transfers. Many species can be grown in rich liquid media intended to mimic the nutritional composition of insect hemolymph (Hajek et al. 2012). This is the simplest form of in vitro culturing but requires stringent aseptic measures as *Entomophthora* fungi in general cannot tolerate antibacterial agents in the media. A further complication is that many isolates do not grow uniformly under these conditions, and liquid-kept cultures may at any one time consist of entomophthoralean cells in a mixture of growth stages including protoplasts, hyphal bodies, and longer hyphal threads with branches (De Fine Licht, pers. obs.; Elya, pers. obs., Fig. 7). It is also possible for some isolates of some species to be grown on rich egg yolk agar media in Petri dishes (Hajek et al. 2012). Such solid-state cultures are also prone to contamination, but may actually induce the formation of conidiophores and conidia (Freimoser et al. 2000). However, in all cases in vitro growth is slow and erratic, which makes it almost impossible to use standard mycological techniques such as obtaining single-spore-isolates or fungal interaction assays. Long-term storage of viable cultures in cryopreservation is possible as exemplified by the Agricultural Research Service Collection of Entomopathogenic Fungal Cultures (ARSEF; USDA, Ithaca, NY), but despite state-of-the-art preservation methods, not all stored *Entomophthora* isolates remain viable (Humber 1994).

Several laboratories have successfully maintained *E. muscae* cultures in vivo by serial transfer between laboratory kept colonies of fly hosts over the years. Providing enough suitable hosts can be steadily supplied, this is feasible but requires a great deal of work to ensure new susceptible hosts can be exposed to sporulating cadavers that, with the generalized life-cycle for *E. muscae* described previously in mind, only are available during short 12–24 h time-windows every other 4–7 d depending on the host-fly *E. muscae* system (De Fine Licht et al. 2017; Elya et al. 2018).

Unlike other entomopathogens commonly studied in laboratory settings, it is virtually impossible to control dosage between individuals. *Entomophthora* conidia are not amenable to suspension in liquid solution because the fragile conidia lyse or otherwise are rendered unviable. Dosage can be roughly controlled by providing a consistent number of fresh cadavers collected prior to the onset of sporulation, but there is still high variation in the number of conidia released from cadaver to cadaver. Dosage can be approximated after the fact, either by placing a glass coverslip in the exposure enclosure in a comparable location to the target host and counting collected conidia after some interval, but this approach is typically not employed as it only provides very rough estimates of the number of spores an insect may have been subjected to.

I*n vivo* cultures can also be initiated or rescued by injecting *E. muscae* cells grown in liquid media into the host insect, but this is very inefficient as only few individuals will succumb and express summit disease followed by fungal sporulation (Carruthers et al. 1985). The site of injection is also important, as injecting grasshoppers into the abdomen with *Entomophaga grylli* did not result in any fungal infections, whereas injection into the dorsal aorta resulted in infection and death of all insects within 12–14 d (MacLeod et al. 1980).

## MOLECULAR AND CELL BIOLOGY

That the molecular and cell biology of the family *Entomophthoraceae* is unusual was already noted in some of the first detailed accounts of the cell cycle of *Empusa aphidis* and *E. sciarae* at the beginning of the twentieth century (Olive 1906). As noted above, the genus name *Empusa* has been synonymized with *Entomophthora* (Hall and Bell 1962), although the two species primarily analyzed by Olive were later shown to belong to *Erynia* (Humber 1982). No detailed analysis of the cell cycle or the size, appearance, mitosis, and number of chromosomes (karyotypology) has since been conducted within *Entomophthora,* whereas scattered records exists for only a few species within other *Zoopagomycota* from the genera *Basidiobolus* (Olive 1907; Sun and Bowen 1972), *Erynia* (Olive 1906; Sawyer 1931; Humber 1975), and *Strongwellsea (*Humber 1975)*;* see Humber (1982) for a review. However, in a direct comparison to *Erynia,* Olive notes that *E. muscae* contains a single large nucleolus within each nucleus (Olive 1906). Several species within *Entomophthora* have multinucleate cells throughout their life-cycle, with as many as 32 nuclei observed in conidiophores of *E. muscae* sensu lato (Keller 1987). In conidiophores, multiple nuclei enter the budding conidium during formation ensuring the continued multinuclearity throughout the life-cycle. The size and number of nuclei within morphologically identical conidia are used as a taxonomic trait to differentiate between members of the *E. muscae* species complex (Keller et al. 1999). The nuclei range in size, and when there are fewer nuclei per cell (e.g., less than 3–7) they can be as large as 5–7 μm (Keller et al. 1999). During growth as hyphal bodies inside insect hosts and when forming conidiophores, the nuclei undergo mitosis. The multiple nuclei within cells do not divide simultaneously and nuclear division is thus independent of the state of division of neighboring nuclei (Olive 1906). The number of chromosomes in *Entomophthora* is unknown, but within the family of *Entomophthoraceae* the basal number of chromosomes appear to be 8, 12, 16 or 32 based on species within *Erynia* and *Strongwellsea (*Humber 1982*)*. Much of what is known about the cell cycle and karyotypology of *Entomophthora* is thus inferred from closely related genera, obviously requiring validation and confirmatory data from *Entomophthora.*

The formation of resting spores is thought to involve the fusion of two hyphal bodies followed by exchange of nuclei, but details on their formation and germination are generally unknown (Keller 2002). It remains unclear if resting spores should be considered sexual zygospores (a product of gametangial conjuction) or asexual azygospores (Humber 2016). It has been suggested that pairwise fusion of nuclei would occur at the time of germination of resting spores, but this has not been confirmed primarily because of difficulty with germinating resting spores in vitro (Macleod 1963). The entire family *Entomophthora*ceae has been suggested to be haploid and homothallic (McCabe et al. 1984; Humber 2012b), i.e., containing two mating types in the same mycelium and thus capable of self-fertilization. This view is primarily based on lack of conclusive evidence for outcrossing with pairings of two different mating types (Humber 2016). For resting spores to undergo classical sexual recombination as zygospores, it in theory requires the fusion of two nuclei in a binucleate zygospore to form a diploid nucleus. Since many fungi in the genus *Entomophthora* have multinucleate cells throughout their life-cycle, including the protoplast stage, hyphal bodies, mycelium, conidia and resting spores, it further complicates unambiguous observations of nuclei fusion and resulting reduction in number of nuclei. Fusion of two nuclei has been observed in *Conidiobolus thromboides (*McCabe et al. 1984*)*, whereas 3–6 nuclei were observed in *E. muscae* resting spores from in vitro cultures (Thomsen et al. 2001). Although not strictly binucleate, this shows a reduction in the number of nuclei in resting spores potentially indicating nuclear fusion.

The notion that resting spores formed via sexual zygospores are the only diploid stage in *Entomophthora* was recently challenged by genome-wide comparative transcriptomic single nucleotide polymorphism (SNP) data, which showed the multiple nuclei within several isolates of *E. muscae* either are likely to consist of two genotype nuclei (i.e., a heterokaryon) present in 50:50 ratios, or that the supposedly haploid nuclei (Humber 2012b), actually are functionally diploid (De Fine Licht et al. 2017). It would be informative for understanding the possibility of outcrossing within *Entomophthora,* to determine whether the two observed genotypes within *E. muscae* isolates reside between nuclei or within nuclei, where the latter could indicate a genome duplication or hybridization event that have lead to functional diploidy (De Fine Licht et al. 2017). The sexual biology of the supposedly haploid and primarily clonally reproducing *Entomophthora* is thus not well resolved, but see Humber (2016) for a recent discussion that predates the recent indication of functional diploidy.

Part of the difficulty with studying these questions in *Entomophthora*, is also because these fungi do not easily lend themselves to modern whole-genome sequencing methodologies (Gryganskyi and Muszewska 2014). First, many of these fungi form “empty” colonies that only grow at the colony edge and contain many empty cells (Batko 1974). This together with their higher activity levels of DNAses and RNAses often result in a low output of highly degraded DNA (Gryganskyi and Muszewska 2014). Second, in addition to the generally unknown genome size, ploidy and karyotypes of most members within *Entomophthoraceae,* the phylum *Zoopagomycota* contains some of the largest fungal genomes ever measured, at 8000 Mb in *Entomophaga aulicae* (a species that with extensive condensed chromatin in the nuclei) (Murrin et al. 1986), and 350–700 Mb for *Basidiobolus* (Henk and Fisher 2012). However, other members of *Zoopagomycota* such as *Conidiobolus coronatus* have a genome size of 39.9 Mb (Chang et al. 2015), similar to the average fungal genome sizes of 10–70 Mb (Gregory et al. 2007). Initial attempts to sequence and assemble a highly contiguous genome within *Entomophthora* have so far been unsuccessful and indicated that the genome of *E. muscae* is very large (> 1,000 Mb) and contains at least 85% repeat content (Elya et al. 2018). This together with unknown ploidy issues of several *E. muscae* isolates (De Fine Licht et al. 2017), makes it a daunting task to tackle these genomes. (De Fine Licht et al. (2016) provide a recent review of *Entomophthora* genetics.) It is tempting to speculate that the obligate insect association within the genus *Entomophthora* coupled with repeated clonal propagation via conidia and potentially almost absent sexual reproduction has resulted in an expansion of repetitive elements that have driven genome size increases. However, genome size estimates for all species other than *E. muscae* within *Entomophthora* are unknown, and it remains to be shown whether the large size of the *E. muscae* genome is representative for the genus.

Compared to the difficulty with obtaining complete genomes, transcriptomic studies of *E. muscae* have fared much better (De Fine Licht et al. 2017; Elya et al. 2018). Based on genome-wide gene expression analyses of the *E. muscae*-fruit fly system, it has for example been shown that the fly host’s immune system recognizes and is activated in response to *E. muscae* infection already after 24 h post-infection (Elya et al. 2018). Earlier microscopical work on *Entomophaga aulicae* had suggested that the absence of many cell-wall epitopes from *Entomophthora* protoplasts effectively prevents the fungi from being recognized, encapsulated and melanized by the host’s cellular immune system (Beauvais et al. 1989). However, *Drosophila melanogaster* genes involved with the humoral immune system were up-regulated early-on after infection (Elya et al. 2018). Similarly, during growth inside the host, *E. muscae* expressed genes involved in nutrient acquisition, such as trehalase (that degrades the most abundant sugar in insect hemolymph, trehalose), patatin (involved in lipid degradation), and an aquaporin (transport of water across membranes) (Elya et al. 2018). Transcriptome data has also revealed that *E. muscae* isolated from house flies has undergone an expansion of trehalase genes compared to soil-living and facultative insect and mammal pathogenic fungus *Conidiobolus coronatus* (De Fine Licht et al. 2017). This supports the hypothesis of an evolutionary transition from a non-entomopathogenic ancestor to the obligate entomopathogenic *Entomophthora* dependent on insect hosts for nutrient acquisition.

**Fig. 8:**
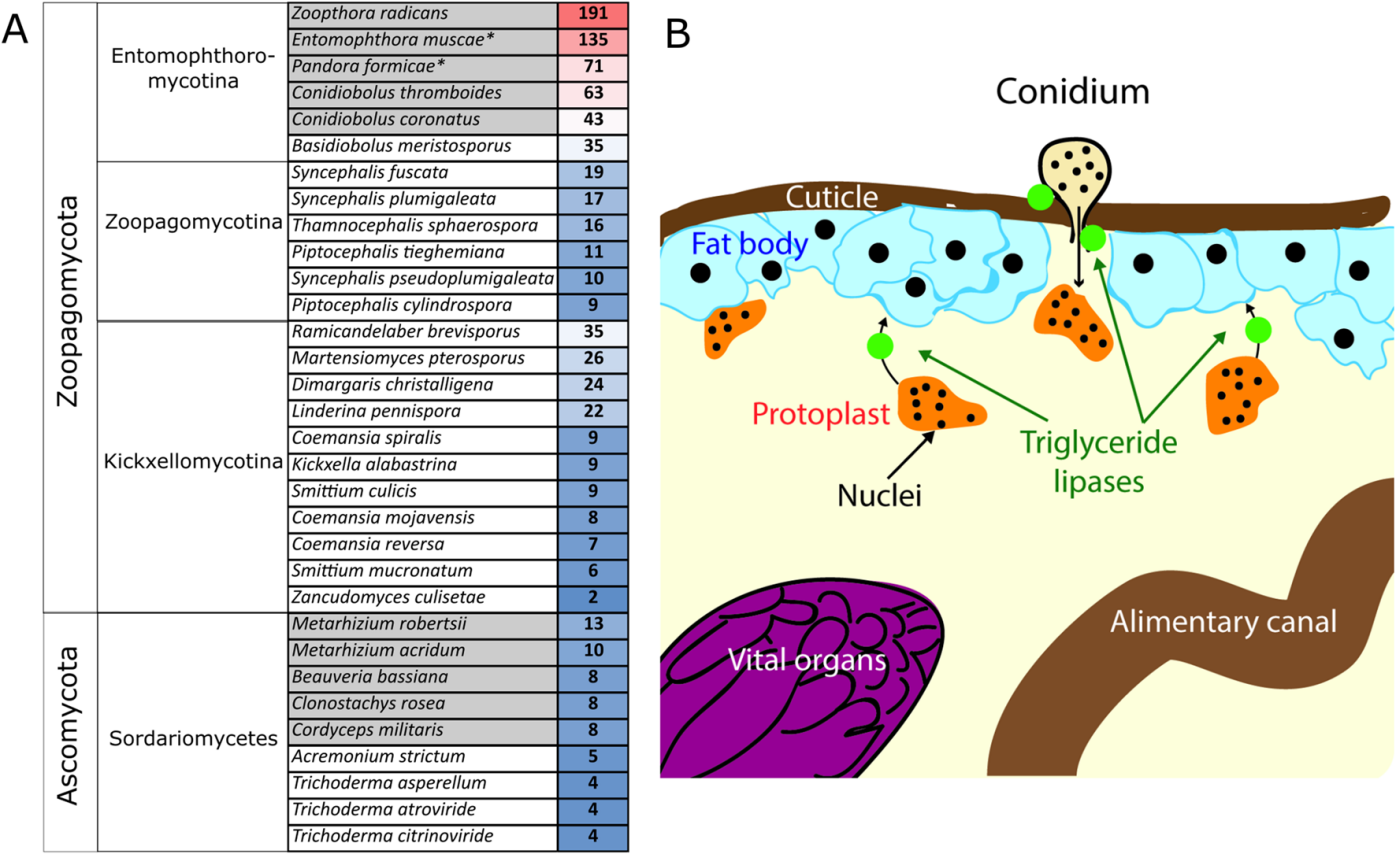
Apparent expansion of triglyceride lipases among entomophthoralean insect–pathogenic fungi. **A** Across the kingdom fungi, individual species vary greatly in the number of triglyceride lipase genes in the genome. Gray boxes denote entomopathogenic fungi within *Zoopagomycota* and *Ascomycota*. Heatmap shows the number of triglyceride lipase genes in the genome with protein family domain (PFAM) PF01764 (Lipase 3) per fungal species. *Data for *E. muscae* and *P. formicae* are not from genomic data but transcriptomic data (Małagocka et al. 2015; De Fine Licht et al. 2017) and all other genomic data are from the Joint Genome Institute Mycocosm database (Grigoriev et al. 2014). **B** Triglyceride lipases are abundant in insect–pathogenic fungi within *Zoopagomycotina* and used when the fungi penetrate the insect cuticle and during consumption of internal fat body tissue

Specific adaptation to insect niches is also seen in the diversity of subtilisin-like serine proteases (SLSPs) within *Entomophthora.* Entomopathogenic fungi use SLSPs to degrade chitin-associated proteins in the insect procuticle when entering through the cuticle. Comparative genomics and transcriptomics analyses revealed that *E. muscae,* together with two other species within order *Entomophthorales* possess a unique group of SLSPs that otherwise is only known from bacteria, *Oomycota* and the early diverging fungi *Cryptomycota*, *Microsporidia*, and now *Entomophthoromycotina* (Arnesen et al. 2018). That the early-diverging insect-pathogenic fungi within *Entomophthorales* show many specific patterns indicative of adaptation to consuming insect tissue is also exemplified by the apparent expansion of triglyceride lipases (Fig. 8). These enzymes appear to be tenfold expanded in *E. muscae* and *Z. radicans* compared to most other *Zoopagomycota* and Asc*o*mycota insect-pathogenic fungi and exemplifies the likely many genomic insights that await discovery in this group.

During growth inside fruit flies, *E. muscae* express two transcripts with homology to white-collar 1, a photoreceptor and a transcriptional regulator of the molecular circadian clock gene *frq*, (Ballario et al. 1996; Lee et al. 2003), and a light-sensitive cryptochrome (Lin and Todo 2005). This intriguingly suggests that *E. muscae* may have the ability to sense light and maintain a molecular clock, which would seem like a prerequisite for expressing the extended phenotype of summit disease. An alternative hypothesis is that the fungus may be manipulating conserved host neuronal networks controlling sleep behavior (Lovett et al. 2020b), which perhaps obviates the need for extensive light and molecular clock-sensing. However, arguably the most fascinating discovery of obtaining these first transcriptomic datasets of *E. muscae* (De Fine Licht et al. 2017; Elya et al. 2018) was the recent discovery of *Entomophthovirus*, an RNA mycovirus of *E. muscae* (Coyle et al. 2018). The virus is a capsid-forming, positive-strand RNA virus in the viral family *Iflaviridae*. Although a single instance of an *E. muscae* isolate without *Entomophthovirus* was reported, the virus seems to be widely and obligately associated with *E. muscae*. The viral family *Iflaviridiae* is almost exclusively comprised of insect vira, which suggests that *Entomophthovirus* has shifted from insects to *Entomophthora* during co-infections inside a dipteran host (Coyle et al. 2018). Curiously, viral particles of a similar size have also been observed in *Strongwellsea magna*, though the authors suggested that this was a baculovirus rather than an iflavirus (Federici and Humber 1977). In the absence of molecular information, the verdict on this tentative identification is still out.

The function of the virus in the interaction with *E. muscae* is not known, but it is tempting to speculate that the virus may help or even make it possible for *E. muscae* to behaviorally manipulate its host (Coyle et al. 2018). Several insect vira are known to behaviorally manipulate their host, such as other members of the *Iflavirus* family that induce a number of behaviors in their insect hosts (Dheilly et al. 2015) or a baculovirus that induce summiting behavior in infected caterpillars (Katsuma et al. 2012). The discovery of this new virus-fungus interaction within *Entomophthora* is a Pandora’s box with numerous open questions relating to the function and obligation of the interactions that has the potential to transform how we view *Entomophthora-*insect interactions.

## EVOLUTION

The close interaction between fungi in the genus *Entomophthora* and their natural insect hosts suggests that the arthropod associated members of the family *Entomophthoraceae* have co-evolved and diversified with insects since their origin 200–400 Mya (Gryganskyi et al. 2012; Boomsma et al. 2014) (Fig. 2). Coevolution between hosts and pathogens occurs when selection by pathogens induces host adaptations that reduce the costs of infection (Janzen 1980). There is no formal evidence of coevolution between *Entomophthora* and their insect hosts (Humber 2008; Gryganskyi et al. 2012), however the presence in certain species of phenotypic traits such as cuticle-breaking conidia germ-tubes, obligate pathogenesis, and behavioral manipulation evidences one-sided adaptation in *Entomophthora* towards host insects as an ecological niche. The close association between *Entomophthora* and insects constitute a basis for potential co-speciation and co-cladogenesis with insects (Gryganskyi et al. 2012, 2013a), but does not require processes of antagonistic coevolution (de Vienne et al. 2013). In order to detect antagonistic coevolution that is due to reciprocal selection, there is a need to focus on phenotypic traits in *Entomophthora* and their natural insect hosts that negatively influence each other such as the presence of mycotoxins and specific insect immune responses. However, it is important to note that such antagonistic coevolution will only influence the traits responsible for the interaction and the genes underlying these traits, not the evolution of either of the interacting species as a whole (Ebert and Fields 2020).

Antagonistic coevolution is generally divided into two overall groups of models: pairwise coevolution (specific coevolution) where one host and one pathogen interact, and diffuse coevolution (unspecific coevolution) where multiple species interact (Ebert and Fields 2020). Although insect pathogenicity as a life-style has evolved numerous times within the kingdom *Fungi* (Humber 2008), there is only scant evidence for coevolution with insects across most of the fungal groups that conquered the insect body. In many cases, and especially for facultative entomopathogens such as the ascomycete genera *Metarhizium* and *Beauveria,* this is likely due to difficulty with detecting weak and/or unclear patterns of unspecific coevolution as these two genera are known to also occur in soil, and interact with plants in the rhizosphere and as endophytes (Barelli et al. 2016; Moonjely and Bidochka 2019). *Metarhizium* and *Beauveria* have received much more research attention than *Entomophthora,* which have likely contributed to the discrepancy between frequently observed patterns of specific coevolution among plant-pathogenic fungi and their host plants and rare to non-existent observed patterns of specific coevolution among Entomopathogenic fungi. Together with the highly host-specific members of the ant-infecting genus *Ophiocordyceps* (Kobmoo et al. 2018), the genus *Entomophthora* is probably one of the most obvious candidates for detecting clear evidence for specific coevolution between entomopathogenic fungi and insects in the future.

## WHERE CAN WE GO FROM HERE?

**Fig. 9:**
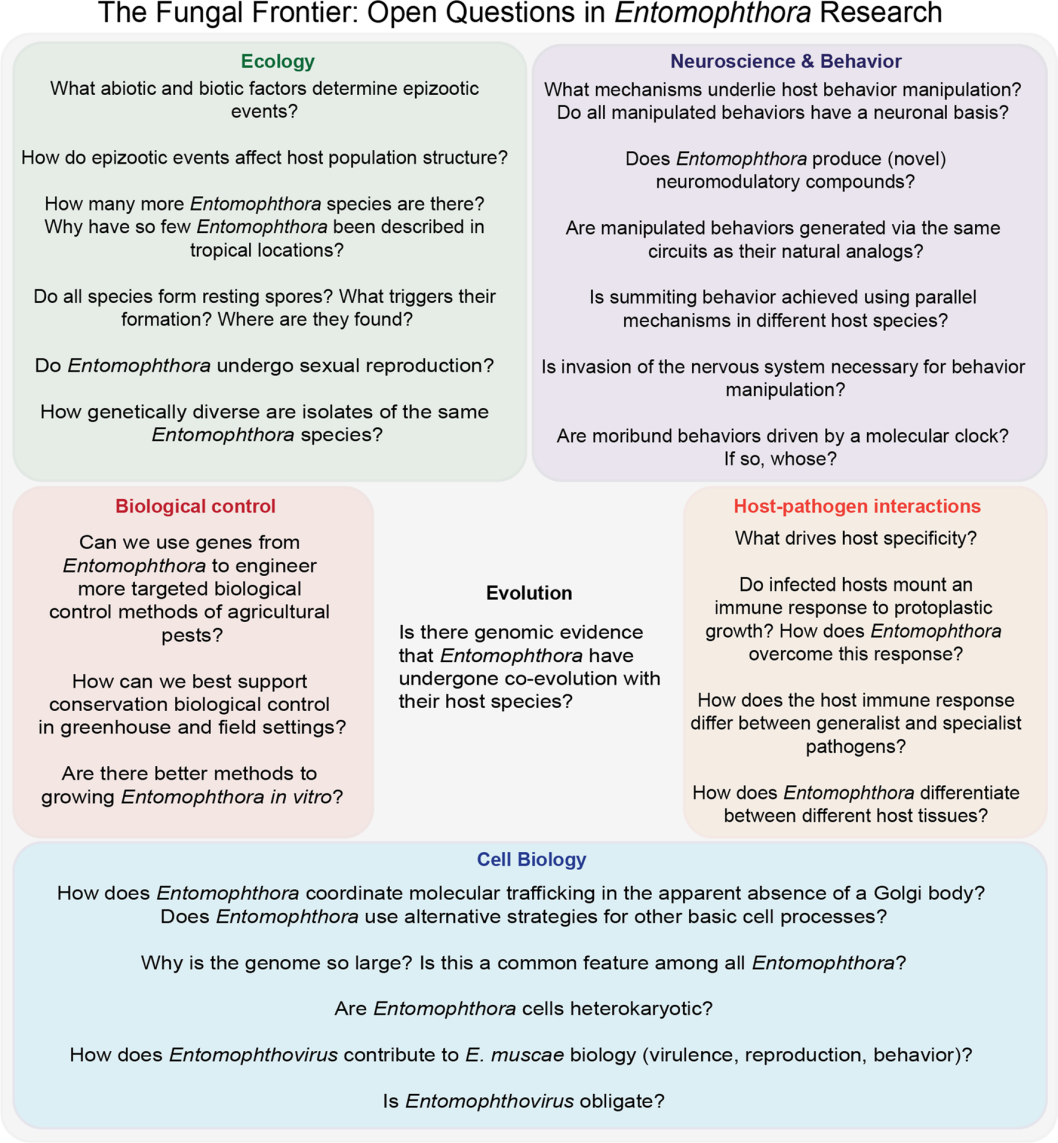
The fungal frontier: open questions in *Entomophthora* research. It should be noted that we have only grouped particular questions under different topics for the purposes of convenience: realistically, all of the questions that the field needs to tackle next span multiple disciplines. Evolution is situated in the middle to represent that it factors into all of the proposed research questions

Over the past 150 years, we have only just scraped the surface of understanding the biology of *Entomophthora*. Now equipped with modern experimental tools, such as improved methods for sequencing large, complicated genomes, and unbiased approaches to studying fungi-host interactions (metabolomics, proteomics, transcriptomics), we are in an exciting time where a whole suite of experiments are possible that were unimaginable only a decade ago. Advancing our understanding of *Entomophthora* biology will lead to key insights into multiple areas of fungal and insect biology, including neurobiology and behavior, immunity, ecology, and evolution. In this final section, we discuss some of the biggest open questions in *Entomophthora* research (Fig. 9).

### Neuroscience and behavior

*Entomophthora* species have honed their ability to elicit specific behaviors in their insect hosts over millions of years of evolution. While work over the last century has led to little, if any, understanding of the mechanistic basis of host behavior modifications by *Entomophthora*, this is now primed to change with the recently-developed *E. muscae-*fruit fly system. Fruit flies boast the most sophisticated neurogenetic tools available for any model organism, meaning that many experiments previously dreamt of are now possible.

Importantly, all the behaviors elicited by *Entomophthora* species are already within the host’s behavioral repertoire; they are just elicited at a particular time in a particular sequence to benefit fungal fitness. Thus, studying how *Entomophthora* turns the proverbial dials of the nervous system offers an opportunity to compare how similar behaviors are created endogenously and ectopically, and learn how the mechanisms giving rise to behavior in each case compare. That is, this work can help us understand how many different ways the same behavior can be produced. In addition, understanding how to generate behavior as outsiders could have far-reaching applications. Fungi are well-known for producing diverse secondary metabolites (Boruta 2018), and there are likely to be neuroactive compounds produced by *Entomophthora,* some of which may be novel and could serve as new tools in research or even, eventually, medicine.

*Entomphthora* fungi target diverse insect species, but many drive summiting behavior. Furthermore, summiting behavior is also elicited by distantly-related fungal pathogens such as particular *Ophiocordyceps* spp*.* as well as some viruses and trematodes (Carney 1969; Andersen et al. 2009; Hoover et al. 2011). Several candidate effectors for behavior manipulation have been identified in *Ophiocordyceps* spp., including a gene cluster predicted to produce an aflratrem-like compound, genus-specific enterotoxins, and species-specific small secreted proteins (de Bekker et al. 2015, 2017; Will et al. 2020). Additionally, two baculovirus genes (*egt* and *ptp*) have been shown to mediate climbing behavior and enhanced locomotion in some baculovirus-larval systems (Hoover et al. 2011; Katsuma et al. 2012; van Houte et al. 2012).If *Entomophthora* spp. convergently evolved similar way(s) to achieve summiting, such common mechanism(s) would reveal conserved principles of behavior encoding across insects, and by extension, across animals. The opposite possibility is equally fascinating: if *Entomophthora* spp. employ unique ways to achieve summiting, this indicates that there was immense evolutionary pressure to evolve this extended phenotype, and could reveal multiple ways of arriving at the same behavior outcome.

### Immunology and insect–pathogen interactions

The extremely high prevalence of *Entomophthora muscae,* at 50–90% infections in cow-stable house fly populations in late summer, demonstrates that not only old or weakened hosts are attacked in the wild (Skovgård and Steenberg 2002). Generalist entomopathogenic fungi with wide host ranges primarily target old or weakened hosts, which would never build up such high prevalence, whereas host-specific entomopathogenic fungi with narrow host ranges such as *E. muscae* can more easily infect all members of a host population (Boomsma et al. 2014). This could superficially indicate that the fungus is “winning” the host-immune-response *vs*. pathogen arms race and leads to the question of why this fungus is still so devastating to flies? Depending on host prevalence and fungal transmission efficiency, pathogens may evolve to become less virulent over time (Ewald 1987; Arnold et al. 2009). However, while *E. muscae* is fatal for the individual infected fly, the relatively long incubation time of 4–7 d from exposure to death allows the infected host to lay eggs and mate during the first few days of infection; this may indicate a limited reduction in overall life-time host fitness. In nature, house flies have an estimated lifespan of about three weeks (Reed and Bryant 2000), so if infection occurs 7–14 d since eclosion, then a reduced life-span due to *E. muscae* infection is perhaps not too substantial. Furthermore, a high *E. muscae* prevalence of > 90% is well above average; a much lower *E. muscae* prevalence in natural populations is much more typical. There is clearly much work to be done towards improving our understanding of *E. muscae*’s natural disease dynamics and epidemiology.

Much of what is known about the general insect immune response towards fungal pathogens is based on generalist and opportunistic fungal infections created using unnaturally high doses in the laboratory (Lu and St Leger 2016). The high host-specificity of species of *Entomophthora,* and perhaps among *E. muscae* isolates in particular, allows exploration of specific host immune responses to host-specific fungal insect pathogens. There is already some evidence that the *D. melanogaster* host immune response towards generalist ascomycete insect–pathogenic fungi is different from the immune response towards *E. muscae* (Wang et al. 2020), but further disentangling the specific immune patterns may potentially reveal completely novel parts of the insect immune repertoire towards fungal pathogens.

An important aspect of host–pathogen interactions occurs between fungal cell-surface epitopes and cell-wall residues and the host immune cells. How fungal morphogenesis and the different growth forms at the various stages of infection influence host immune recognition and response is largely unknown. For example, how *E. muscae* undergoes morphological transitions from appressoria-like penetration cells, to wall-less protoplasts, to hyphal bodies, and eventually mycelial threads and conidiophores, is not well understood. Is there a division of labor between the different fungal growth structures in overcoming the host immune system and facilitating competition for host nutrients? Which fungal cells secrete or induce the behavioral manipulation? Some *E. muscae* cells, for instance, occur in and around the brain following infection (Elya et al. 2018), while protoplasts consume fat bodies in the abdomen, but it is not known if these differentially localized cell populations serve different roles in the course of infection. Many of these questions could begin to be addressed by employing spatially resolved metabolomics and transcriptomics methods.

### Cell biology

Fungi in the genus *Entomophthora* are estimated to be approximately as evolutionarily distant from fungi in the *Ascomycota* and *Basidiomycota* as humans are from *Cnidaria* (the phylum of jellyfish and sea anemones), approximately 820 My (Kumar et al. 2017). *Entomophthora* is still ~ 500 Mya diverged from *Conidiobolus*, arguably the most well-studied entomophthoralean entomopathogenic fungus, which is comparable to when humans and sharks last shared a common ancestor (Kumar et al. 2017). As a result of this long divergence time, entomophthoralean fungi may well have developed novel strategies for some basic life processes.

For example, *Entomophthora* has been reported to lack a Golgi apparatus, an organelle that is present in virtually all other eukaryotic cells (Latgé et al. 1988). We currently have no idea as to why *Entomophthora* lacks this organelle and how it achieves the functions that the Golgi normally serves (e.g., trafficking proteins and lipids to the correct part of the cell). Another curiosity is that *E. muscae* has recently been found to have a close relationship with an *Iflaviridae*-related virus, a family of viruses that is normally only encountered in insects. It remains to be seen what role this virus plays in *E. muscae* biology: is it essential for cell survival? Does it contribute to virulence? Does it play a role in behavior manipulation? And furthermore, do other entomophthoralean fungi have similar viral infections?

Another mysterious feature of *Entomophthora* is the extraordinarily large genome. At present, we can only guess at the reason for this. Though conventional wisdom holds that parasitic genomes tend to become more streamlined over time, this expansion of non-coding DNA appears to parallel a similar trend in plant pathogenic fungi, where the exclusively biotrophic and more host-specific plant pathogens have larger genomes and more repetitive elements (Raffaele and Kamoun 2012). This increased genome size has been posited to be adaptive for these pathogens in providing flexibility to keep up in the constant arms race with the host plant immune system. Perhaps this is also true for *Entomophthora* and neighboring Entomophthorales. The acquisition of additional *Entomophthora* and entomophthoralean genomes, perhaps by leveraging third generation long-read sequencing technology and new linked-read methods, would provide the comparative dataset needed to begin addressing this hypothesis.

### Biological control

Much research effort into *Entomophthora* has been driven by a desire to use these fungi as highly specific biological control agents towards certain pest insects. However, difficulty with mass production of infectious spores in vitro and the viable formulation and storage into an easily applicable commercial product has halted their direct use in classical biological control (Vega et al. 2012). Conservation biological control where the environment or agricultural practices are altered in such a way to improve conditions for naturally occurring or released entomophthoralean biological control agents appear to have had the most success (Eilenberg et al. 2001; Tobin and Hajek 2012). However, if we can better understand the mechanisms of how these fungi are able to attract, infect, and appear year after year in natural insect populations, the molecular and chemical insights might allow currently unknown chemicals, proteins or virulence factors produced by these fungi to be artificially produced with biotechnological methods and built into new biological control measures.

### Ecology

Much fundamental knowledge about *Entomophthora* populations is unknown. For example, the abiotic and biotic factors influencing when and where epizootics occur are unclear. Also, the degree of genetic variation in natural *Entomophthora* populations is not known for most species, as we do not even know how many species there are. In the few studies that have performed detailed sampling of *E. muscae* over an extended period of time, genetic variation was ample and the population was subdivided into what could be considered host-ecotypes (Gryganskyi et al. 2013b). How extended genetic variation is maintained in populations is an open question. How much can be attributed to local host adaptation? How does the degree of clonal *vs*. potential sexual reproduction via the formation of zygospores influence genetic population structure within *Entomophthora* species? When sampling *E. muscae* in house fly populations in cow stables, each barn seems to harbor a single clonal lineage of *E. muscae* with frequent exchange between barns (Lihme et al. 2009), but how much exchange of *E. muscae* occurs between house fly populations with increasing geographic distance is unclear. It is also unclear whether the same clonal lineage reappears in each cow stable year after year. Overall, there is a need to understand many fundamental questions of *Entomophthora* population dynamics, both in natural and managed ecosystems. These issues could begin to be addressed by working with citizen scientists to “crowd-source” the observations of *Entomophthora* epizootics both to collect new isolates and select additional field sites.

## CONCLUSION

*Entomophthora* species live among us and are frequently encountered, yet many aspects of their fundamental biology remain mysterious. Here, we have endeavored to provide a comprehensive summary of our understanding of their biology, including host range and specificity, geographical and temporal observances, life-cycle, molecular and cell biology, and evolution. With twenty-first century tools, including low-input and long-read sequencing, untargeted -omic methods, and online citizen science resources, the study of *Entomophthora* can move beyond descriptive work and more fully into the molecular era to begin to address the many open questions in the field (Fig. 9).

## Supplementary Information

**Additional file 1**. Excel spreadsheet containing source data for Figure 4. Sheet named "Entomophthora" lists all instances of Entomophthora sightings, including source of sighting information ("database"), date of sighting ("observed_on"), geographical coordinates where sighted ("latitude", "longitude"), and species ("scientific_name"). Sheet named "Diptera" lists all instance of Dipterans sightings from iNaturalist with same column headers as "Entomophthora" sheet.

**Additional file 2**. Matlab code employed to read in Additional File 1 and generate Figure 4.

## Data Availability

Not applicable.

## Abbreviations

AHT: Active host transmission
ARSEF: USDA Agricultural Research Reservice Collection of Entomopathogenic Fungal Cultures
CA: Corpora allata
CT: Cadaver transmission
ITS: Internal transcribed spacer
PAMP: Pathogen associated molecular pattern
PFAM: Protein family domain
PRR: Pattern recognition receptor
RFLP: Restriction fragment length polymorphism
RAPD: Random amplified polymorphic DNA
SLSP: Subtilisin-like serine protease

## Footnotes

1 This review follows the IMA Fungus convention (laid out in Thines et al. 2020) to italicize all Latin names to facilitate their identification in the text.

2 Despite a genus of orchids already named *Empusa* and some scientists immediately decrying its invalidity (Fresenius 1856), a subset of the field continued to use *Empusa* to refer to a fungal genus within the *Entomophthorales* until at least the 1950s (Macleod 1963).

3 This species was originally reported as *E. muscae* but was later re-identified as *E. schizophorae* by Keller (1987).
